# Thioredoxin *h*2 and *o*1 Show Different Subcellular Localizations and Redox-Active Functions, and Are Extrachloroplastic Factors Influencing Photosynthetic Performance in Fluctuating Light

**DOI:** 10.3390/antiox10050705

**Published:** 2021-04-29

**Authors:** Liang-Yu Hou, Martin Lehmann, Peter Geigenberger

**Affiliations:** Department Biology I, Ludwig-Maximilians-University Munich, 82152 Planegg-Martinsried, Germany; martin.lehmann@biologie.uni-muenchen.de (M.L.); geigenberger@biologie.uni-muenchen.de (P.G.)

**Keywords:** Trx *o*1, Trx *h*2, metabolomics, ascorbate, glutathione, NAD(P)(H), photosynthetic performance, fluctuating light, *Arabidopsis thaliana*

## Abstract

Arabidopsis contains eight different *h*-type thioredoxins (Trx) being distributed in different cell organelles. Although Trx *h*2 is deemed to be confined to mitochondria, its subcellular localization and function are discussed controversially. Here, cell fractionation studies were used to clarify this question, showing Trx *h*2 protein to be exclusively localized in microsomes rather than mitochondria. Furthermore, Arabidopsis *trxo1*, *trxh2* and *trxo1h2* mutants were analyzed to compare the role of Trx *h*2 with mitochondrial Trx *o*1. Under medium light, *trxo1* and *trxo1h2* showed impaired growth, while *trxh2* was similar to wild type. In line with this, *trxo1* and *trxo1h2* clustered differently from wild type with respect to nocturnal metabolite profiles, revealing a decrease in ascorbate and glutathione redox states. Under fluctuating light, these genotypic differences were attenuated. Instead, the *trxo1h2* double mutant showed an improved NADPH redox balance, compared to wild type, accompanied by increased photosynthetic efficiency, specifically in the high-light phases. Conclusively, Trx *h*2 and Trx *o*1 are differentially localized in microsomes and mitochondria, respectively, which is associated with different redox-active functions and effects on plant growth in constant light, while there is a joint role of both Trxs in regulating NADPH redox balance and photosynthetic performance in fluctuating light.

## 1. Introduction

Reduction-oxidation reactions serve as fundamental components for the processes of bioenergetics and signal pathways. Proper operation of biosynthetic processes and signal transduction relies on the optimization of redox potential spans. This appears to be of importance for plants since plants often encounter diverse environmental challenges. For example, light intensity in the field changes very rapidly. Such fluctuation of light intensity leads to short-term imbalances in the redox state of NADPH/NADP^+^ and the energy state of adenylated nucleotides and eventually results in the over-production of reactive oxygen species, which dramatically change the cellular redox poise [1,2]. Thus, plants develop several mechanisms to deal with such challenges.

Ascorbate (AsA) and glutathione (GSH) redox systems are known as powerful buffering mechanisms to balance cellular redox states [3]. Both AsA and GSH are able to metabolize hydrogen peroxide (H_2_O_2_). While both systems can work independently, they have also shown to function in an integrated manner [4]. In the AsA-GSH cycle, AsA serves as an electron donor for ascorbate peroxidase (APX) to detoxify H_2_O_2_. Then, AsA is converted into monodehydroascorbate (MDHA), which can be regenerated as AsA with the help of monodehydroascorbate reductase (MDHAR). Part of MDHA is subsequently converted into dehydroascorbate (DHA), which can also be reduced to AsA via the activity of dehydroascorbate reductase (DHAR) using glutathione as electron donors. This would generate oxidized glutathione disulfide (GSSG), which can be regenerated to GSH via the activity of glutathione reductase (GR) using NADPH as an electron donor. The reduced glutathione can then be used for the regeneration of AsA again or the detoxification of H_2_O_2_ [5]. The disruption of the AsA-GSH cycle greatly compromises plant stress tolerance as well as plant development [6,7,8]. Furthermore, there are growing reports indicating an interaction of AsA, GSH and thioredoxin (Trx) systems to maintain the cellular redox balance [9,10,11,12,13]. In this context, plastidial MDHAR was found to be redox-regulated by thioredoxins (Trxs) [14].

Thioredoxins are ubiquitous proteins in various organisms. Plants have the most elaborate Trx system, with respect to different reduction pathways and isoforms being involved in the redox regulation of widespread metabolic processes in different subcellular compartments [4]. In Arabidopsis, the 20 Trx isoforms are categorized into seven types: Trx *f*, *h*, *m*, *o*, *x*, *y* and *z* [15,16]. The *f*, *m*, *x*, *y* and *z*-type Trxs are plastidial proteins and responsible for regulating redox balance and metabolic processes in chloroplasts, e.g., enzyme activation of Calvin-Benson cycle, carbon metabolism [17,18], the regulation of malate valve [19], meristem development, cyclic electron transport [20,21,22], the regulation of antioxidation systems [23,24,25,26], and plastidial gene expression [27].

The *o*-type Trxs comprise two isoforms, Trx *o*1 and *o*2. The Trx *o*1 protein is specifically localized in mitochondria, with the exception of pea (*Pisum sativum*), where this protein was found in both mitochondria and nuclei [28,29]. Work with Arabidopsis knock-out mutants and biochemical studies indicate diverse functions of Trx *o*1, including the regulation of stress responses and cell cycle progression, as well as the activation of tricarboxylic-acid-cycle (TCA) enzymes and alternative oxidase (AOX) in respiratory processes of mitochondria [30,31,32,33,34], even though it is still under debate whether Trx *o*1 is involved in the redox regulation of AOX [35]. Furthermore, the Trx *o*1 protein has been suggested to be involved in the regulation of NAD(P)(H) redox state and the inhibition of mitochondrial glycine decarboxylase (GDC) L-protein and thus, be able to fine-tune photorespiratory processes in Arabidopsis [36].

The *h*-type Trxs are the largest Trx family, which contains eight isoforms in Arabidopsis. Many of *h*-type Trxs, including Trx *h*1, *h*3, *h*4, and *h*5, were found to be localized in cytosol, while Trx *h*7 and *h*8 were reported to be associated with ER-Golgi membrane compartments and Trx *h*9 was found to be localized at the plasma membrane [28,37,38,39]. As the largest Trx family, the *h*-type Trxs were found to have diverse functions. In barley and wheat, Trx *h* is involved in the seed developmental processes [40,41]. In *Brassica*, there is a role of Trx *h* in the self-incompatibility response [42,43]. In Arabidopsis, it has been reported that Trx *h*3 functions as a molecular chaperone to help plants overcome heat stress [44], while Trx *h*5 is strongly induced in response to pathogen infection and oxidative stresses to regulate these responses [45,46]. Furthermore, Trx *h*9 plays a role in intercellular communication [37]. In contrast to the other *h*-type Trxs, Trx *h*2 was found to harbor regulatory functions in several metabolic pathways of mitochondria. The first report from Daloso et al. indicated Trx *h*2 to cooperate with Trx *o*1 to regulate TCA cycle enzyme activities [33]. With the analyses on *trxh2* Arabidopsis mutants, Trx *h*2 was found to be involved in photorespiratory processes via redox-regulating the activity of the mitochondrial GDC L-protein [47]. However, the subcellular localization of Trx *h*2 remained unclear. Using the transient expression of GFP-fused Trx *h*2 protein in onion epidermal cells, Meng et al. suggested that the Trx *h*2 is localized in both cytosol and mitochondria [37], while Traverso et al. used the same approach to propose that this protein is associated with the ER-Golgi membrane system [38]. 

The studies mentioned above suggest that Trx *h*2 is likely to share redundant functions with Trx *o*1 in mitochondria, while it remains unclear whether Trx *h*2 is indeed localized within this organelle. In the present work, we used cell fractionation studies as an additional technique to clarify the subcellular localization of the Trx *h*2 protein and confirmed its exclusive localization to the microsomal fraction rather than to mitochondria. To further understand the role of Trx *h*2 in comparison to mitochondrial Trx *o*1, we selected two Arabidopsis T-DNA insertion mutants (*trxo1* and *trxh2*), showing deficiencies of Trx *o*1 and Trx *h*2, respectively. Both lines have been comprehensively characterized in previous studies [33,45] and therefore serve as representative T-DNA insertion mutants for the following analyses. Both lines were further crossed to generate a double mutant (*trxo1h2*) showing joint deficiencies of both Trxs. In experiments to directly compare these lines, single deficiencies of Trx *o*1 and Trx *h*2 led to different effects in maintaining the redox states of AsA and GSH as well as growth in medium light, while joint deficiencies of Trx *o*1 and Trx *h*2 led to additive effects in balancing NADP(H) redox state and photosynthetic efficiency in fluctuating light. This indicates different roles of Trx *o*1 and *h*2 depending on the light conditions.

## 2. Materials and Methods

### 2.1. Plant Material

#### 2.1.1. Arabidopsis T-DNA Insertion Mutant Lines

The Arabidopsis wild type, Columbia-0 (Col-0), two representative T-DNA insertion mutants, *trxh2* (SALK-079516) [45] and *trxo1* (SALK-042792) [33], and one crossed double mutant, *trxo1h2*, were used for this study. Both *trxh2* (SALK-079516) and *trxo1* (SALK-042792) have been comprehensively characterized in previous studies [33,45] and therefore serve as representative T-DNA insertion mutants. Both lines were crossed to generate a double mutant (*trxo1h2*) showing joint deficiencies of both Trxs.

#### 2.1.2. Establishment of Trx *h*2 Overexpression Line

The coding region of Trx *h*2 gene was amplified using the primers, TRXH2-F_GW (5′-aaaaagcaggctttatgggaggagctttatcaactgtg-3′), TRXH2-R_GW (5′-agaaagctgggtttgctctgagtttgctaactttcttc-3′), attB1 adapter (5′-ggggacaagtttgtacaaaaaagcaggct-3′) and attB2 adapter (5′-ggggaccactttgtacaagaaagctgggt-3′). The DNA fragment was first subcloned into pDONR221 (Invitrogen, ThermoFisher Scientific, Waltham, MA, USA) and then transferred into pJCV52 (VIB, Ghent, Belgium) using a GATEWAY cloning kit (Invitrogen, ThermoFisher Scientific, Waltham, MA, USA). Arabidopsis plants were transformed by following the floral dip method [48]. The transformed plants were grown on the medium containing 50 mM of kanamycin. The survived plants were used to propagate seeds for the selection of homozygous transgenic lines. 

#### 2.1.3. Growth Conditions

All plants were grown under constant medium light (ML) or fluctuating light (FL). The light intensity of ML was set as 150 μmoL m^−2^ s^−1^ with a 16-h-light/8-h-dark regime. Plants were grown under ML for three weeks, and the entire rosette leaves were harvested at the end of the night (EN) and the end of the day (ED). The setup of FL contained loops of 5 min low light (LL, 50 μmoL m^−2^ s^−1^) and 1 min high light (HL, 500 μmoL m^−2^ s^−1^) with a 12-h-light/12-h-dark regime. Plants were grown under FL for four weeks, and the entire rosette leaves were harvested during the LL and HL phases in the middle of the day. The growth temperature was maintained at 22 °C for both conditions.

### 2.2. Cell Fractionation

#### 2.2.1. Microsome Isolation

The microsome isolation was performed by using the kit (Minute^TM^ Plant Microsomal Membrane Extraction Kit; Invent Biotechnologies, Plymouth, MA, USA). In brief, 200 mg of fresh plant leaves were homogenized in 300 μL of cold buffer A and then transferred into a filter cartridge followed by centrifugation for 20 min at 14,000× *g* in 4 °C. The supernatant was kept on ice for later cytosolic fraction isolation, and the pellet was re-suspended in 300 μL of cold buffer B followed by centrifugation for 10 min at 11,000× *g* in 4 °C. The supernatant was then transferred into a 2 mL microtube with the supplement of 1 mL of one-time strength PBS. The mixture was centrifuged for 30 min at 14,000× *g* in 4 °C. The supernatant was removed, and the pellet was kept on ice for later use.

#### 2.2.2. Cytosolic Fraction Isolation

The cytosolic fraction isolation was performed via ultracentrifugation, as described before [49]. In brief, the supernatant mentioned above was first diluted in 2.5 mL of PBS buffer, and the whole extract was centrifuged for 30 min at 100,000× *g* in 4 °C. Afterward, the pellet was discarded, and the supernatant was also kept on ice for later use.

#### 2.2.3. Mitochondrion Isolation

The mitochondrion isolation was performed as described before [50] with modifications. In brief, 30 g of 2-week-old seedlings was homogenized in 250 mL of extraction medium (300 mM sucrose 1.5 mM EDTA; 15 mM MOPS; 0.4% (*w/v*) fatty acid-free BSA; 0.6% (*w/v*) PVP-K30; 100 mM AsA; 10 mM DTT; pH 7.4) on ice. The leaf debris was removed using miracloth followed by centrifugation for 5 min at 1500× *g* in 4 °C. The supernatant was transferred into a new tube and centrifuged for 20 min at 16,000× *g* in 4 °C. The pellet was carefully re-suspended in 1 mL of washing medium I (300 mM sucrose; 10 mM TES; 0.1% (*w/v*) fatty acid-free BSA; pH 7.5) and mixed with 250 mL of washing buffer I, followed by centrifugation for 5 min at 1500× *g* in 4 °C. The supernatant was transferred into another tube and centrifuged for 20 min at 16,000× *g* in 4 °C. The pellet was carefully re-suspended in 1 mL of washing medium I and kept on ice in the dark for later use. The separation buffer was made of heavy gradient solution (7 mL of 2-times strength washing medium I; 3.9 mL of Percoll; 3.1 mL of 20% (*w/v*) PVP-K30) and light gradient solution (7 mL of 2-times strength washing medium I; 3.9 mL of Percoll; 3.1 mL of double-distilled water) using a gradient maker. Re-suspended pellet was carefully transferred onto the top of the separation buffer followed by centrifugation with slow acceleration and disengaged brake for 40 min at 27,000× *g* in 4 °C. The purified mitochondria were transferred into a new tube and washed with 40 mL of washing medium II (300 mM sucrose; 10 mM TES; pH 7.5) followed by centrifugation for 15 min at 16,000× *g* in 4 °C. The mitochondrial pellet was kept on ice for later use.

#### 2.2.4. Protein Extraction and Immunoblotting Analyses

Above samples were first re-suspended in the protein extraction buffer (20 mM Tris-HCl, pH 7.0, 5 mM EDTA, 100 mM NaCl, 0.5% (*v/v*) Triton X-100, 1% (*w/v*) SDS, 6 M urea, 2 M thiourea) and then mixed with Laemmli buffer [51]. The protein extract was incubated at 85 °C for 10 min, followed by centrifugation for 5 min at 20,000× *g* at 25 °C. Ten microliters of supernatant was applied into a 4–20% SDS acrylamide gradient gel for electrophoresis, then transferred onto a 0.45 μm PVDF membrane for immunoblotting analyses. The antibody dilution factors are listed below: thioredoxin *h*2 (Trx *h*2), 1:250; alternative oxidase (AOX), 1:1000; cytosolic fructose 1,6-bisphosphatase (cyt-FBPase), 1:2500; calnexin (CNX): 1:5000. The Trx *h*2 antibody was produced by ThermoFisher Scientifc (Waltham, MA, USA), and the others were purchased from Agrisera. 

### 2.3. Molecular Characterization

#### 2.3.1. Total RNA Extraction 

Fifty milligrams of ground leaf sample was suspended in 500 μL of RNAzol reagent (Sigma-Aldrich, St. Louis, MO, USA) and then mixed with 100 μL of ice-cold chloroform followed by the centrifugation for 10 min at 20,000× *g* in 4 °C. The upper aqueous phase was transferred into a new tube followed by the addition of 300 μL of isopropanol and centrifugation for 10 min at 20,000× *g* in 4 °C. The precipitated RNA pellet was suspended in 200 μL of nuclease-free water followed by the addition of 200 μL of PCI reagent (phenol/chloroform/isoamylalcohol, 25:24:1 (*v*/*v*/*v*)). The mixture was centrifuged for 10 min at 20,000× *g* in 4 °C, and the upper phase was transferred into a new tube followed by the addition of 20 μL of 3 M sodium acetate (pH 5.2) and 500 μL of absolute ethanol. The whole mixture was centrifuged for 10 min at 20,000× *g* in 4 °C. The precipitated RNA pellet was washed with 70% (*v/v*) ethanol and dehydrated in the hood. Afterward, the pellet was dissolved in 50 μL of nuclease-free water. The concentration was determined by a spectrophotometer (NanoDrop^TM^ 2000; ThermoFisher Scientific, Waltham, MA, USA).

#### 2.3.2. Reverse Transcription Reaction

Five hundred nanograms of total RNA was incubated with the mixture containing 2 μL of 5-times reaction buffer and 0.5 μL of iScript reverse transcriptase (Biorad, Hercules, CA, USA) and supplied with sufficient nuclease-free water to make up 10 μL in total volume. The reactions were performed in the thermocycler (C1000 Touch^TM^ Thermal Cycler; Biorad, Hercules, CA, USA) with the program: 25 °C for 10 min, 42 °C for 30 min and 85 °C for 5 min. The cDNA sample was diluted 10 times with nuclease-free water prior to the real-time quantitative PCR.

#### 2.3.3. Real-Time Quantitative PCR

Five microliters of the cDNA sample was mixed with the reaction mixture containing 10 μL of 2-times SYBG reagent (Biorad, Hercules, CA, USA), 0.5 μL of 10 μM forward primer, 0.5 μL of 10 μM reverse primer and 4 μL of nuclease-free water. The reaction was carried out in the thermo cycler (iQ5 Multicolor Real-Time PCR Detection System; Biorad, Hercules, CA, USA) with the program: 95 °C for 1 min, and 40 cycles, each containing 95 °C for 30 s, 60 °C for 30 s, and 72 °C for 30 s. For building up the melting curve of primers, the temperature started at 55 °C and gradually elevated to 95 °C in 0.5 °C increments. The gene expression was quantified followed the 2^−ΔΔCt^ method [52,53]. The primer sequences were listed in the following: TRXh2-F (5′-catgccatggctgataagttcaatg-3′), TRXh2-R (5′-tcaagttcgtcctttttggcacc-3′), TRXo1-F (5′-gcctggtgtggaccatgcag-3′), TRXo1-R (5′-cagtgttggcacagccgtgat-3′), EF1α-F (5′-tgagcacgctcttcttgctttca-3′) and EF1α-R (5′-ggtggtggcatccatcttgttaca-3′).

### 2.4. Metabolite Profiling

#### 2.4.1. Total Metabolite Extraction

Fifty milligrams of ground leaf material was suspended in the extraction buffer containing 340 μL of ice-cold methanol, 10 μL of ribitol solution (0.2 mg mL^−1^) and 10 μL of ^13^C-sorbitol (0.2 mg mL^−1^), followed by the incubation on ice for 30 min. The extract was further mixed with 200 μL of chloroform and 400 μL of water followed by the centrifugation for 15 min at 25,000× *g* in 4 °C. Fifty microliters of upper aqueous phase was transferred into a glass vial and dehydrated using a vacuum at ambient temperature.

#### 2.4.2. Gas Chromatography Coupled Time-of-Flight Mass Spectrometry

The detection of metabolite followed the method published before [54,55,56]. The details of sample preparation and condition were also described in the previous publication [57]. The results were analyzed using ChromaTOF 4.5 and TagFinder 4.1 software [58].

### 2.5. Measurement of Metabolites

#### 2.5.1. Ascorbate and Dehydroascorbate

The assay followed the protocol described before [59]. In brief, 20 mg of pulverized leaf material was suspended in 200 μL of HCl solution (0.2 M) with vortex for 4 min followed by centrifugation for 10 min at 16,000× *g* in 4 °C. Two hundred microliter of supernatant was transferred to a new microtube followed by the addition of 20 μL of NaH_2_PO_4_ solution (0.2 M, pH 5.6) and a sufficient amount of NaOH solution (0.2 M) to make the pH value stay between five and six. The extract was kept on ice until further use. For the measurement of AsA, 20 μL of the extract was mixed with 100 μL of NaH_2_PO_4_ solution (0.2 M, pH 5.6) as well as 75 μL of double-distilled water, and the optical density at 265 nm was recorded every minute using the spectrophotometer (FilterMax F5; Molecular Device, San José, CA, USA). When the reaction was stable, 2 μL of AsA oxidase (50 U mL^−1^) was applied into the mixture to initiate the reaction. The decrease of optical density at 265 nm represents the AsA amount. For the measurement of total ascorbate, 30 μL of the extract was pre-incubated with 3 μL of DTT (25 mM) and 42 μL of NaH_2_PO_4_ solution (0.12 M) for 30 min in 25 °C. Twenty microliters of this mixture was used for the measurement as above. Subtracting the reduced AsA amount from total ascorbate yields the amount of DHA. The pure AsA and DHA were used to prepare the standard solution for generating the calibration curve. 

#### 2.5.2. Glutathione and Glutathione Disulfide

The extraction followed the same approach mentioned above. For the measurement of total GSH, 10 μL of the extract was mixed with 130 μL of reaction buffer (154 mM NaH_2_PO_4_, pH 7.5; 15.4 mM EDTA; 0.77 mM NADPH; 0.92 mM DTNB; 0.2 U GSH reductase) and 60 μL of double-distilled water. The optical density at 412 nm was recorded every 30 s using the spectrophotometer (FilterMax F5; Molecular Device, San José, CA, USA). The reaction slope was used for the following calculation. For the measurement of GSSG, 120 μL of the extract was pre-incubated with 2 μL of 2-vinylpyridine for 30 min in 25 °C to complex free GSH followed by centrifugation for 5 min at 20,000× *g* in 25 °C. Ten microliters of the supernatant was used for the measurement as above. Subtracting double amounts of GSSG from total glutathione yields the amount of reduced GSH. The pure GSH and GSSG were used to prepare the standard solution for generating the calibration curve.

#### 2.5.3. Pyridine Nucleotides

The assay followed the protocol described before [60]. The details of sample preparation and detection were performed as described in the previous publication [57].

### 2.6. Pulse-Amplitude-Modulation Measurement

#### 2.6.1. Constant Medium Light Treatment

Three-week-old plants grown under medium light were placed in the dark for 30 min prior to the measurement and shifted into the image PAM instrument (MAXI version; WALZ, Effeltrich, Germany). The program was set as below: 20 min for constant medium light (150 μmoL m^−2^ s^−1^), followed by 10 min for the dark. The florescence signals were recorded every 30 s.

#### 2.6.2. Fluctuating Light Treatment

Four-week-old plants grown under fluctuating light were placed in the dark for 30 min prior to the measurement and shifted into the image PAM instrument. The program was set as below: 4 cycles of fluctuating light containing loops of 5 min low light (50 μmoL m^−2^ s^−1^) and 1 min high light (500 μmoL m^−2^ s^−1^), and 5 min for the dark. The florescence signals were recorded every 30 s.

#### 2.6.3. Calculation

The yield of photosystem II, Y(II), was calculated via the formula listed below: Y(II) = (F_m_’ − F)/F_m_’. F, fluorescence yield measured briefly before application of a saturation pulse; F_m_’, maximal fluorescence yield of illuminated sample with all PSII centers closed.

### 2.7. Statistical Analysis

The Graphpad Prism 8.0 software was used to generate figures and carry out statistical analyses. The differences between genotypes were assayed via ANOVA followed by Dunnett’s post hoc test unless otherwise described. The R program was used to generate a heatmap of metabolite profile and perform principal component analysis.

### 2.8. Accession Numbers

Sequence data of this article is available from GeneBank/EMBL database. *Arabidopsis thaliana* Trx *h*2: At5g39950, Trx *o*1: At2g35010.

## 3. Results

### 3.1. The Trx h2 Protein Is Localized in the Microsomal Fraction

Unlike the mitochondrial Trx *o*1, the subcellular localization of Trx *h*2 remains controversial. While previous studies used transient expression systems and reporter genes, here, a cell fractionation approach was alternatively adopted to clarify this debating issue. 

**Figure 1:**
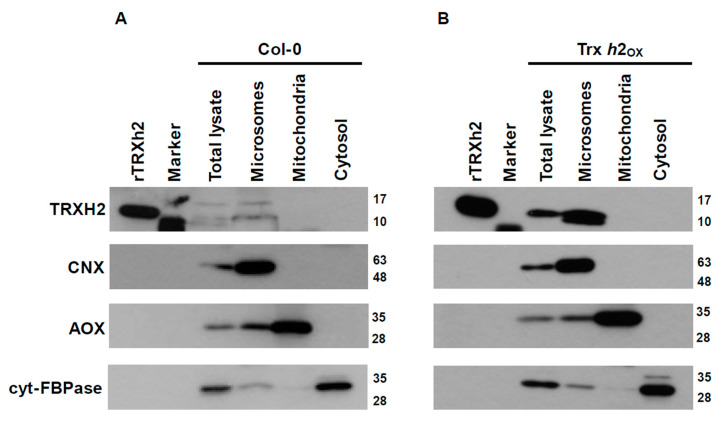
The enrichment of the Trx *h*2 protein in different subcellular fractions. The wild-type plants (Col-0) (**A**) and overexpression line (Trx *h*2_ox_) (**B**) were grown under medium light condition for two weeks and harvested for sample preparation and cell fractionation. The detection of the TRXH2 protein and other subcellular protein markers was performed via immunoblot analysis. Recombinant Trx *h*2 protein (rTRXh2) served as positive control; calnexin (CNX) served as microsomal marker; alternative oxidase (AOX) served as mitochondrial marker; cytosolic fructose-1,6-bisphosphatase (cyt-FBPase) served as cytosolic marker. In the lane labeled “Marker,” marker proteins from the protein ladder were loaded. The numbers next to each blot indicate the molecular weight (kDa) of protein markers.

Figure 1 shows the Trx *h*2 protein level in different subcellular fractions enriched in microsomes, mitochondria and cytosol, documented by immunoblots using calnexin (CNX), alternative oxidase (AOX) and cytosolic fructose-1,6-bisphosphatase (cyt-FBPase) as marker proteins, respectively. The fractionation assays were performed using plant material from the wild type and a Trx *h*2 overexpression line (Trx *h*2_ox_), showing increased Trx *h*2 expression by 100 times of wild-type level (Figure S1A,B). The Trx *h*2 protein was clearly present in the purified microsomal fractions of wild-type (Figure 1A) and Trx *h*2_ox_ plants (Figure 1B), which were enriched in CNX, while no Trx *h*2 signals were detectable in purified mitochondria (enriched in AOX) and purified cytosolic fractions (enriched in cyt-FBPase). These results clearly and unequivocally demonstrate that the Trx *h*2 protein is confined to microsomes rather than to mitochondria or cytosol. 

### 3.2. The Trxh2 and Trxo1 Mutants Show Differential Growth Phenotypes When Grown in Different Light Conditions

**Figure 2:**
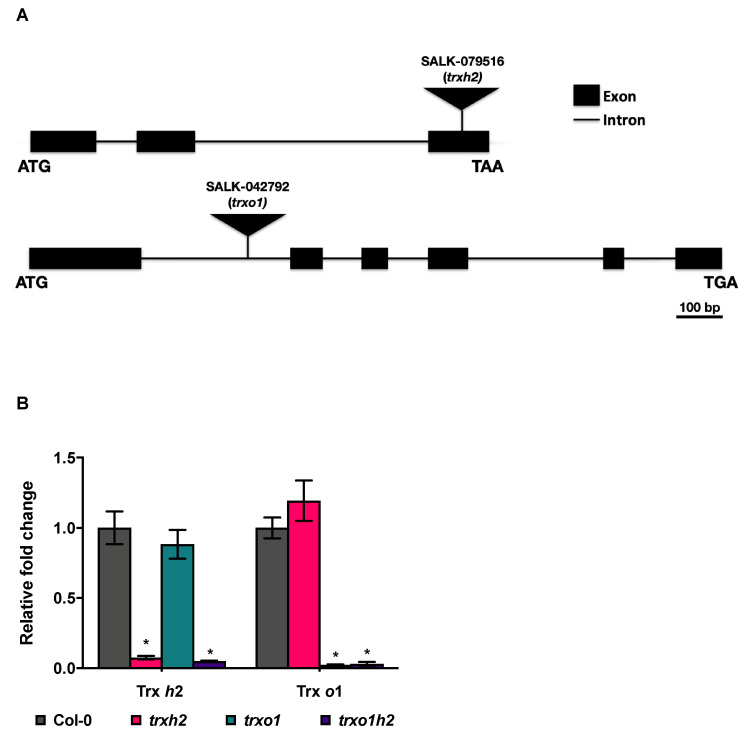
Molecular characterization of *trxh2*, *trxo1* and *trxo1h2*. (**A**) The scheme of T-DNA insertion sites in *trxh2* and *trxo1* mutants. (**B**) The transcript levels of Trx *h*2 and Trx *o*1 in mutant lines compared to the wild type (Col-0). Mean values and standard errors derived from 6 biological replicates. The statistical analyses were performed using ANOVA and the Dunnett’s test (* *p* < 0.05, in comparison to the wild type).

Since Trx *h*2 showed a different subcellular localization in comparison to Trx *o*1, we next investigated whether both proteins show differential functions. To do this, we analyzed *trxo1* [33] and *trxh2* [47] T-DNA insertion lines, which were shown to be representative lines in previous studies, together with a double mutant (*trxo1h2*) generated by crossing of these lines. In confirmation to previous studies on the *trxo1* [33] and *trxh2* [47] T-DNA insertion lines, the T-DNA inserted at the third exon of the Trx *h*2 gene in *trxh2*, while the T-DNA inserted at the first intron of the Trx *o*1 gene in *trxo1* (Figure 2A). Furthermore, the expression of the Trx *h*2 gene in *trxh2* significantly decreased by 95% compared to the wild type, and the expression of the Trx *o*1 gene in *trxo1* significantly decreased by 98% compared to the wild type. In the double mutant (*trxo1h2*), the expression of both Trx *h*2 and *o*1 was significantly decreased in comparison to the wild type (Figure 2B). This documents that these three different T-DNA insertion lines are null mutants and appropriate for the following applications.

**Figure 3:**
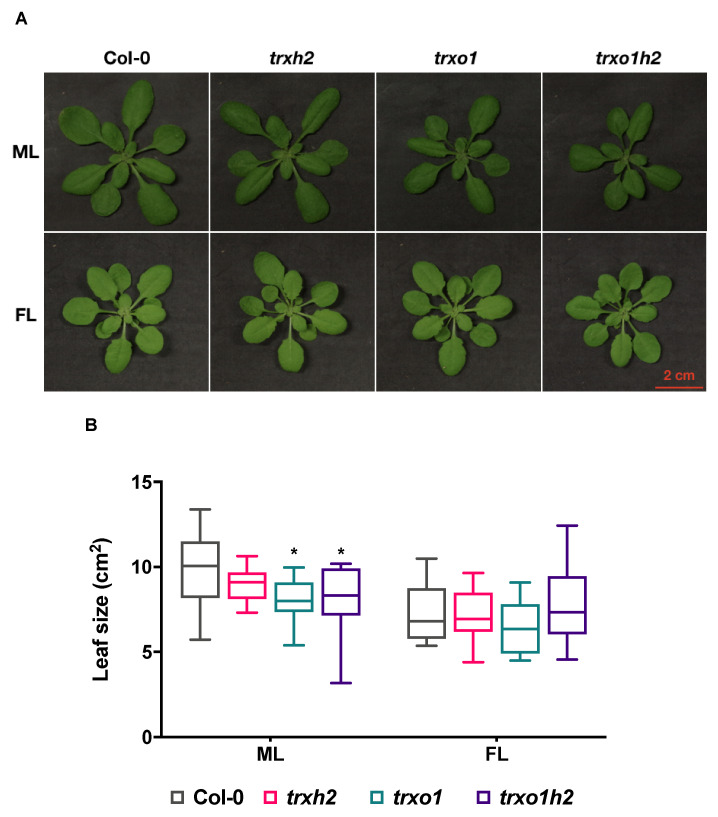
Growth phenotype of the wild type (Col-0) and mutant lines (*trxh2*, *trxo1* and *trxo1h2*) grown under medium (ML) or fluctuating (FL) light conditions. (**A**) Visible phenotype of rosette leaves. (**B**) The box plot of leaf size. Mean values derived from 10–14 plants. The statistical analyses were performed by using ANOVA and the Dunnett’s test (* *p* < 0.05, in comparison to the wild type). Scale bar = 2 cm.

To understand whether the deficiency of Trx *h*2 and *o*1 affects plant growth, the growth phenotype of the mutant lines was analyzed. In addition to a standard growth condition with medium-light intensity (ML), plants were also grown under fluctuating light intensity (FL), consisting of a loop of five-minute low-light phase and one-minute high-light phase, to mimic natural light conditions in the field. Under constant ML conditions, the growth of the *trxh2* mutant was comparable to the wild type, while *trxo1* single and *trxo1h2* double mutants showed retarded growth (Figure 3A). Surprisingly, this growth phenotype was lost under FL conditions, in which all mutants grew like the wild type (Figure 3B). There is a general decrease in plant growth when FL is compared with constant ML conditions (Figure 3A,B), with the decrease being less strongly expressed in *trxo1* and *trxo1h2* mutants. These data show that deficiency of Trx *o*1 leads to impaired plant growth under normal light conditions but not in fluctuating light, while deficiency of Trx *h*2 has no effect on growth in any of the conditions. 

### 3.3. Nocturnal Metabolite Levels of Trxh2, Trxo1 and Trxo1h2 Mutants Cluster Differently to the Wild Type

**Figure 4:**
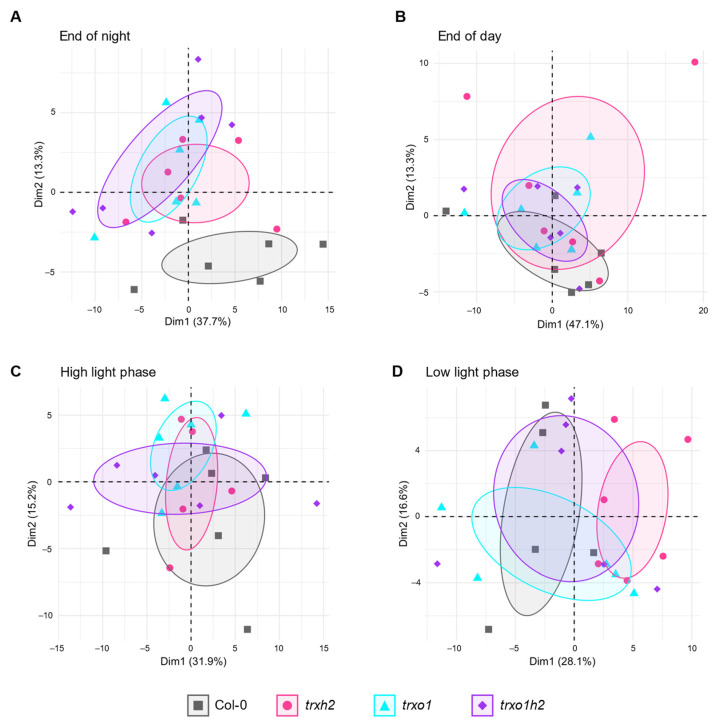
The principal component analysis on metabolite profile of the wild type (Col-0) and the mutant lines (*trxh2*, *trxo1* and *trxo1h2*). Arabidopsis plants grown under medium light conditions were harvested at the end of the night (**A**) and the end of the day (**B**), respectively. Arabidopsis grown under fluctuating light conditions were harvested at the high-light phase (**C**) and low-light phase (**D**), respectively. The samples were used for metabolite profiling via GC-TOF-MS, and the results were used for principal component analysis by using R software. Dim 1: 1st principal component; Dim 2: 2nd principal component.

To further understand the differential effects of Trx *h*2 and *o*1 on plant growth, a GC-TOF-MS approach was performed to analyze metabolite profiles in the mutants under different light conditions. We first analyzed the data set using principal component analysis (PCA) to get a global pattern of metabolite changes. When nocturnal metabolism was investigated, the three mutants clustered differently to the wild type. The *trxo1* and *trxo1h2* mutants showed a similar cluster, while the *trxh2* single mutant was only overlapping partly with the *trxo1* mutant (Figure 4A). Interestingly, when metabolism was analyzed in the light, the PCA shows a similar cluster for the wild type and mutants. This holds true for the day phase of constant ML conditions (Figure 4B), as well as for the high-light (HL) and low-light (LL) phases of FL conditions (Figure 4C,D). This indicates that Trx *h*2 and Trx *o*1 are both important for nocturnal metabolism, with partially overlapping functions.

**Figure 5:**
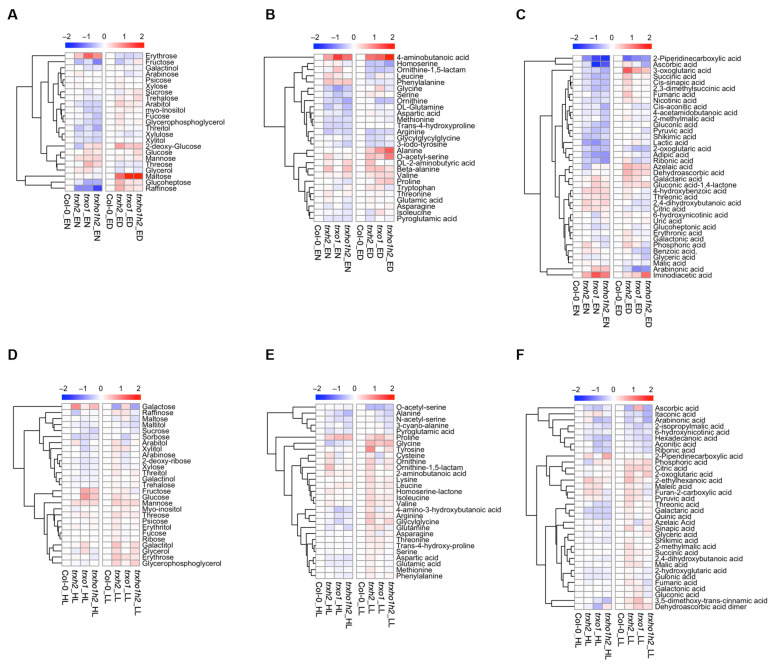
Metabolite profile of the wild type (Col-0) and the mutant lines (*trxh2*, *trxo1* and *trxo1h2*). Arabidopsis plants grown under medium light conditions (**A**–**C**) were harvested at the end of the night (EN) and the end of the day (ED), respectively. Arabidopsis grown under fluctuating light conditions (**D**–**F**) were harvested at the high-light phase (HL) and low-light phase (LL), respectively. The samples were used for metabolite profiling via GC-TOF-MS. (**A**,**D**), Sugars and sugar alcohols. (**B**,**E**), Amino acids. (**C**,**F**), Organic acids and TCA cycle intermediates. Results are normalized to Col-0 with log_2_ transformation and visualized as a heatmap with hierarchical clustering done by R software. Data are taken from Tables S1 and S2.

Indeed, at the end of the night, the *trxh2* and *trxo1* single mutants and the *trxo1h2* double mutants showed a mild decrease in the levels of several soluble sugars, which were the cases for fructose (51–84% of wild-type level), glucoheptose (64–88% of wild-type level), raffinose (37–54% of wild-type level) and xylulose (81–88% of wild-type level), and amino acids, which were the case for arginine (76–84% of wild-type level), glutamine (71–88% of wild-type level), glycine (61–78% of wild-type level), methionine (85% of wild-type level), ornithine (63–80% of wild-type level) and serine (73–89% of wild-type level). Notably, in the mutant lines, the levels of 4-aminobutanoic acid (GABA) and phenylalanine were significantly increased when compared to the wild type (Figure 5A,B, left panel; Table S1). In the mutant lines, there was also a clear decreasing pattern in the levels of many organic acids, including 2-piperidinecarboxylic acid (30–54% of wild-type level), adipic acid (56–71% of wild-type level), gluconic acid (68–88% of wild-type level), lactic acid (55–69% of wild-type level), ribonic acid (57–76% of wild-type level), pyruvic acid (71–82% of wild-type level), 2-oxoglutaric acid (69–82% of wild-type level) and succinic acid (67–81% of wild-type level; Figure 5C, left panel; Table S1). Surprisingly, joint deficiencies in Trx *h*2 and *o*1 had no additive effects on metabolite accumulation. However, these metabolite changes in the mutants versus wild type were not sustainable when metabolism in the light was analyzed (Figure 5A–C, right panel; Table S1). 

Under FL conditions, deficiency of Trx *h*2 and *o*1 had minor effects on the accumulations of most sugars and sugar alcohols (Figure 5D). In the HL phases, the changes of most amino acids in the mutant lines were also very subtle, except for alanine (69–92% of wild-type level), glycine (75–91% of wild-type level) and proline (1.3–1.5 times wild-type level; Figure 5E, left panel, Table S2). However, in the LL phases, several amino acids showed significant changes in either the single or double mutants. This included glycine (1.3–1.5 times wild-type level), proline (1.2–1.4 times wild-type level) and O-acetyl-serine (68–76% of wild-type level; Figure 5E, right panel, Table S2). In the HL phases, some organic acids showed a decreasing tendency in the *trxo1* and *trxo1h2* mutants, which were the cases for galataric acid (80% of wild-type level), hexadecanoic acid (71–85% of wild-type level), ribonic acid (77% of wild-type level) and threonic acid (80% of wild-type level), while, in the LL phases, the changes were not sustainable (Figure 5F; Table S2). Taken together, the results indicate the significance of Trx *h*2 and *o*1 in nocturnal metabolism, while there were only minor effects in FL conditions.

### 3.4. Deficiencies in Trx h2 and o1 Differentially Affect the Reduction States of Ascorbate and Glutathione

**Figure 6:**
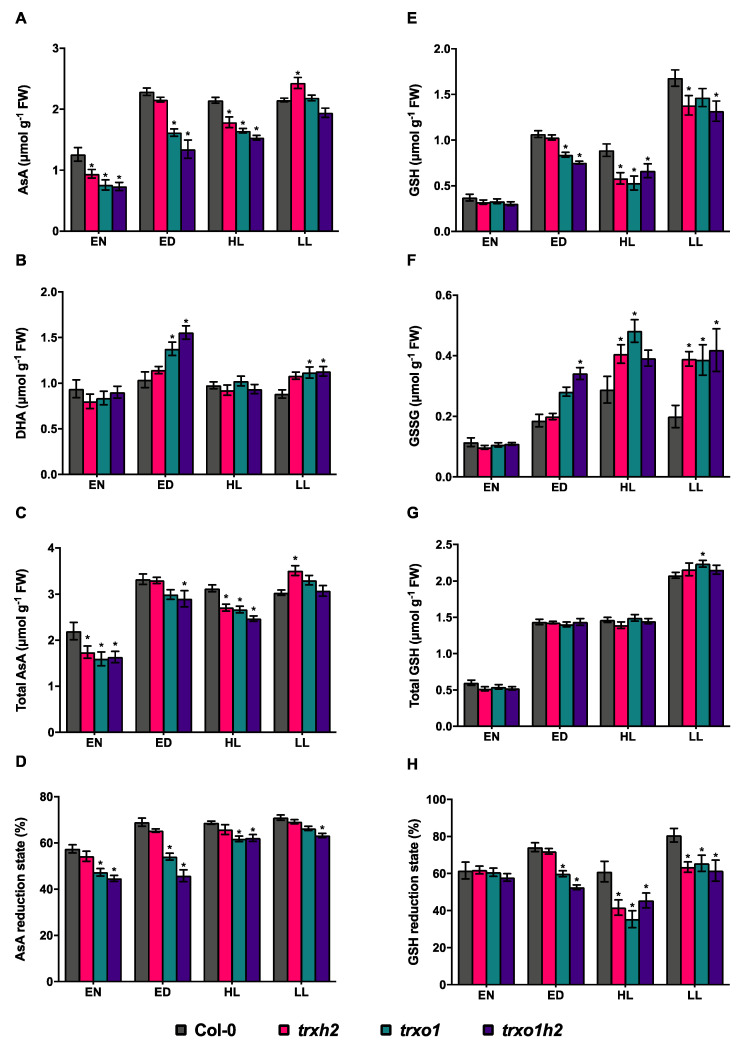
The redox couples of ascorbate (AsA) and glutathione (GSH) in the wild type (Col-0) and the mutant lines (*trxh2*, *trxo1* and *trxo1h2*). Arabidopsis plants grown under medium light conditions were harvested at the end of the night (EN) and the end of the day (ED), respectively. Arabidopsis grown under fluctuating light condition were harvested at the high-light phases (HL) and low-light phases (LL), respectively. (**A**) The level of ascorbate. (**B**) The level of dehydroascorbate (DHA). (**C**) The level of total AsA. (**D**) The reduction state of AsA. (**E**) The level of reduced GSH. (**F**) The level of oxidized glutathione (GSSG). (**G**) The level of total GSH. (**H**) The reduction state of GSH. Mean values and standard errors derived from 6 biological replicates. The statistical analyses were performed using ANOVA and the Dunnett’s test (* *p* < 0.05, in comparison to the wild type).

To understand whether Trx *h*2 and *o*1 are involved in the maintenance of cellular redox status, the reductive states of ascorbate (AsA)/dehydroascorbate (DHA) and reduced glutathione (GSH)/oxidized glutathione (GSSG) redox couples were analyzed. In the wild type, the total levels of AsA, DHA (Figure 6A–C), GSH and GSSG (Figure 6E–G), as well as the AsA and GSH reductive states (Figure 6D,H) were higher in the different light conditions, compared to dark. When focusing on AsA system first, all three mutants showed a decrease in total AsA levels in the dark and HL phases, while *trxo1* single and *trxo1h2* double mutants also showed a decrease in ML conditions, compared to the wild type (Figure 6A,C). In addition to this change, the AsA reduction state (calculated as the AsA/(AsA + DHA) ratio in %) in mutant lines except the *trxh2* single mutant was lower than the wild type under all analyzed conditions (Figure 6D). The decrease in AsA reduction state due to knockout of Trx *o*1 was more strongly expressed in the dark and ML than in FL conditions. The joint deficiencies of Trx *o*1 and *h*2 did not lead to stronger effects compared to Trx *o*1 single deficiency.

Looking at the GSH system, all three mutants showed similar decreases in GSH levels (Figure 6E) and GSH redox states (Figure 6H; calculated as the GSH/[GSH+GSSG] ratio in %) and an increase in GSSG level (Figure 6F) under FL conditions (HL and LL) compared to the wild type, and similar patterns of the three parameters in ML were observed in the *trxo1* single and *trxo1h2* double mutants, but not in the *trxh2* single mutant. In contrast to this change, GSH levels and GSH redox states were similar in all genotypes in the dark. The levels of total GSH pool in all three mutant lines were comparable to the wild type under all analyzed conditions (Figure 6G). These results indicate that both Trx *h*2 and *o*1 serve as positive regulators in maintaining the reductive state of the GSH system specifically in FL conditions; however, Trx *o*1, but not Trx *h*2, regulates the reductive state of the AsA system under all analyzed conditions. This shows that Trxs *h*2 and *o*1 regulate the AsA and GSH redox systems in a different manner. 

### 3.5. Deficiencies in Trx h2 and o1 Affect the NADPH Redox State in Fluctuating Light

**Figure 7:**
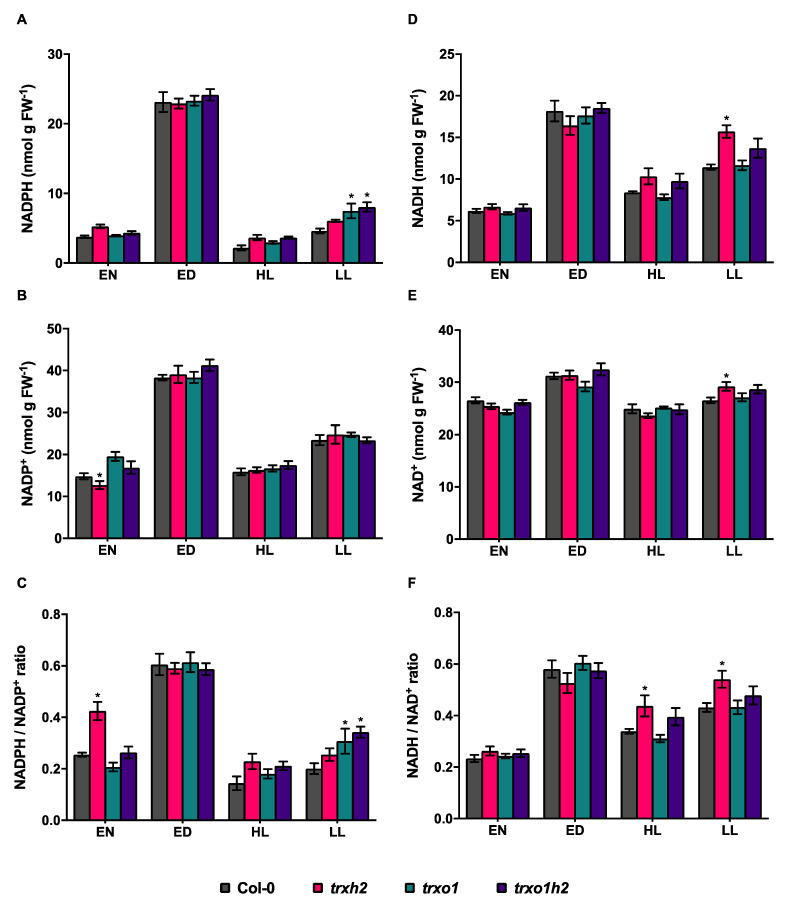
The redox couples of NADP(H) and NAD(H) in the wild type (Col-0) and the mutant lines (*trxh2*, *trxo1* and *trxo1h2*). Arabidopsis plants grown under medium light conditions were harvested at the end of the night (EN) and the end of the day (ED), respectively. Arabidopsis grown under fluctuating light conditions were harvested at the high-light phases (HL) and low-light phases (LL), respectively. (**A**) The level of NADPH. (**B**) The level of NADP^+^. (**C**) The NADPH-to-NADP^+^ ratio. (**D**) The level of NADH. (**E**) The level of NAD^+^. (**F**) The NADH-to-NAD^+^ ratio. Mean values and standard errors derived from 6 biological replicates. The statistical analyses were performed using ANOVA and the Dunnett’s test (* *p* < 0.05, in comparison to the wild type).

The redox couples, NADPH/NADP^+^ and NADH/NAD^+^, are also important components for maintaining the cellular redox balance. They also serve as substrates for Trx reductases to regulate the redox state of Trxs. To understand whether deficiencies of Trxs *h*2 and *o*1 affect the redox balance of NAD(P)(H), the levels of NADPH, NADP^+^, NADH and NAD^+^ were analyzed (Figure 7). In the wild type, the levels of NADPH (Figure 7A) and NADH (Figure 7D), as well as the NADPH/NADP^+^ (Figure 7C) and the NADH/NAD^+^ ratios (Figure 7F), were much higher in the light than in the dark, when plants were analyzed under constant ML conditions. This confirms previous studies showing a strong increase in the reduction states of the NADPH and NADH systems upon illumination under normal growth conditions [19,61]. Interestingly, the light-induced increase in the NADPH/NADP^+^ ratio was wiped out under FL intensities, yielding NADPH and NADP^+^ levels in HL and LL phases that were below those reached in the night (Figure 7C). Additionally, the light-induced increase in the NADH/NAD^+^ ratio was strongly attenuated in FL, compared to ML (Figure 7F).

In all three mutants, the levels of NADPH, NADP^+^, NADH and NAD^+^, as well as the NADPH/NADP^+^ and NADH/NAD^+^ ratios, were similar to wild-type levels when plants were analyzed under ML conditions. However, one exception is the increase in the NADPH/NADP^+^ ratio in the *trxh2* single mutant (Figure 7C), which was not reflected by a corresponding increase in the NADPH level (Figure 7A). Since the NADPH/NADP^+^ ratio remained at the wild-type level in the *trxo1h2* double mutant, we do not think that this effect is related to a more specific function of Trx *h*2. 

Interestingly, there were increases in NADPH level (Figure 7A) and NADPH/NADP^+^ reduction state (Figure 7C) in mutants relative to the wild type when plants were analyzed in FL intensities. The increase in these parameters was more markedly in LL compared to HL phases of FL and was more strongly expressed in the *trxo1* single and *trxo1h2* double mutants than in the *trxh2* single mutant. In the LL phases of FL, the trxo1h2 double mutant with joint deficiencies of Trx o1 and h2 reached higher NADPH level and NADPH/NADP+ ratios compared to the other genotypes.

Taken together, while illumination with constant ML intensity led to increased reduction states of the NADP(H) and NAD(H) systems, these redox couples remained in a more oxidized state upon illumination with FL intensities. Interestingly, the FL-induced oxidation of the NADP(H) system was attenuated by the joint deficiencies in Trx *h*2 and *o*1, specifically in the LL phases of FL. These results indicate that both Trxs have negative effects on the levels and reduction states of NADPH in FL environments.

### 3.6. Joint Deficiencies in Trx h2 and o1 Lead to Enhanced Photosynthetic Efficiency in Fluctuating Light

**Figure 8:**
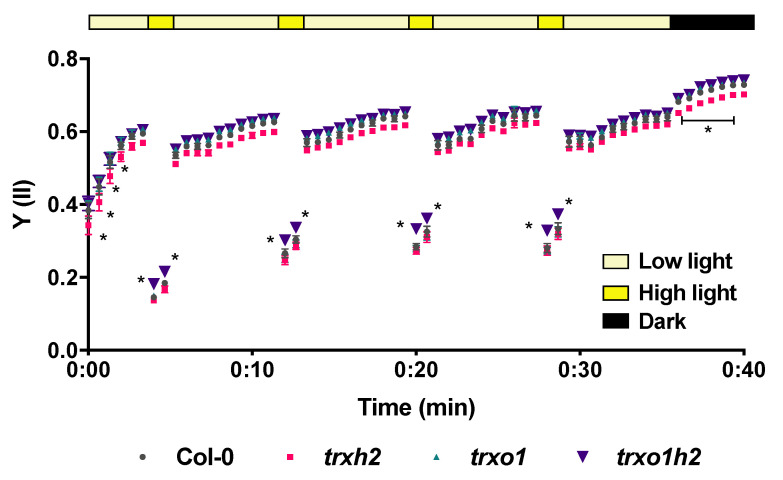
The quantum yield of photosystem II (Y(II)) in the wild type (Col-0) and the mutant lines (*trxh2*, *trxo1* and *trxo1h2*) in fluctuating light. Arabidopsis plants were grown in a fluctuating light environment for four weeks. Chlorophyll fluorescence kinetics during the alternating periods of low light (5 min) and high light (1 min) were recorded by using a PAM system, and the values were used for calculating Y(II). Mean values and standard errors derived from 6 biological replicates. The statistical analyses were performed using ANOVA and the Dunnett’s test (* *p* < 0.05, in comparison to the wild type).

The effects of Trxs *h*2 and *o*1 on the NADPH reduction state in FL might be associated with changes in photosynthesis. Therefore, a pulse-amplitude-modulation (PAM) approach was used to analyze the photosynthetic performance of the mutants relative to the wild type. When plants grown in FL were analyzed, the quantum yield of photosystem II (Y(II)) decreased dramatically in HL versus LL phases of FL. This decrease in Y(II) was attenuated in the *trxo1h2* double mutant, showing a significantly higher Y(II) in the HL phases of FL, compared to the wild type or single mutants (Figure 8). In contrast to these changes in FL, the *trxo1h2* double mutant revealed only minor effects on Y(II) when plants were grown under constant ML conditions (Figure S2). Under steady-state conditions of constant ML and dark, Y(II) levels of all mutants were similar to the wild-type level. However, during dark–light transitions, the *trxo1h2* double mutant showed a faster increase in Y(II) kinetics (Figure S2) compared to the wild type or single mutants. These results indicate that Trxs *h*2 and *o*1 are jointly involved in the control of photosynthetic performance, both in FL environments and during rapid dark-light transitions, but have no sustainable effects on photosynthesis under steady-state conditions in constant ML.

## 4. Discussion

Arabidopsis contains eight different *h*-type Trxs, which are distributed to different cell organelles. Although Trx *h*2 has been proposed to be localized specifically to mitochondria and to fulfill similar roles as mitochondrial Trx *o*1, its subcellular localization and function are still a matter of debate. In this work, we used a cell fractionation approach to clarify the localization of Trx *h*2. To further understand the functions of Trx *h*2 and *o*1, we performed a series of physiological and biochemical analyses to directly compare representative *trxh2* and *trxo1* single mutants as well as their crossed double mutant in different light conditions. Our results show that Trx *h*2 is exclusively localized to the microsomes rather than to mitochondria. In constant light, Trx *h*2 and *o*1 harbor differential functions on plant growth, nocturnal metabolism and redox states of AsA and GSH, while in fluctuating light, both types of Trxs jointly influence NADP(H) redox states and photosynthetic efficiency.

### 4.1. Thioredoxin h2 Is Associated to ER/Golgi Showing a Different Subcellular Localization in Comparison to Trx o1 

The plant Trx *h* family consists of various isoforms, with Arabidopsis containing eight different *h*-type Trxs. Since the encoded proteins were found to have no obvious transit peptides, they were initially anticipated to be confined to the cytosol [39]. Interestingly, in further studies using transient expression systems and reporter genes, Trx *h*2 was proposed to localize to subcellular compartments other than the cytosol, but the results were not consistent. Studies by Gelhaye et al. and Meng et al. in poplar (*Populus trichocarpa*) and Arabidopsis proposed Trx *h*2 to reside in mitochondria [37,62], while Traverso et al. used similar studies in Arabidopsis to document that this protein is associated with the endoplasmic reticulum (ER)-Golgi membrane system [38]. To clarify the subcellular localization of Trx *h*2, we used a cell-fractionation study combined with immunoblot analyses in Arabidopsis. By analyzing both wild-type and Trx *h*2_ox_ plants, we found that the Trx *h*2 protein is clearly enriched in microsomal fractions, while it is not detectable in purified mitochondria or cytosolic fractions. This provides unequivocal and direct biochemical evidence that Trx *h*2 is confined to the endomembrane system rather than to mitochondria or cytosol (Figure 1). Our cell fractionation studies further confirm the GFP reporter studies published by Traverso et al. [38] and are in line with Trx *h*2 being absent in Arabidopsis mitochondrial proteome databases [63]. Moreover, the Trx *h*2 protein was found to contain an N-terminal extension that harbors a myristoylated residue, which is known to target cytosolic proteins to ER/Golgi membranes [38]. When the myristoylable Gly at the N-terminal extension of Trx *h*2 was substituted by Ser, the modified Trx *h*2 was found to be relocated to the cytosol, providing evidence that the N-myristoylation motif is responsible for inducing the ER/Golgi localization of Trx *h*2 [38]. As a myristoylated protein, Trx *h*2 is probably anchored to the cytoplasmic face of the Golgi/ER, indicating metabolic functions in the cytosol rather than characteristic ER functions, like protein synthesis and folding. More studies will be necessary to confirm this interpretation.

### 4.2. Thioredoxins h2 and o1 Have Different Roles in Regulating Growth, ASA-GSH Redox States and Metabolite Profiles in Non-Stressed Conditions

To investigate whether the different subcellular localization relates to different functions of Trxs *h*2 and *o*1, we analyzed *trxh2* and *trxo1* single mutants together with a *trxo1h2* double mutant to allow a direct comparison of the different genotypes with respect to plant growth, cellular redox states, metabolite profiles and photosynthetic parameters under non-stressed ML conditions. Under these conditions, deficiency of Trx *o*1 led to a decrease in plant growth, while Trx *h*2 deficiency had no such effect (Figure 2). Interestingly, a joint deficiency of both Trxs did not lead to additive effects, indicating Trx *o*1 and *h2* to have different roles in growth regulation. The retardation of plant growth was not due to a decrease in photosynthetic efficiency since the quantum yield of PS II was similar in all genotypes in ML (Figure S2). 

The cellular redox status, which includes different redox systems, is important for metabolic regulation, antioxidant function and signaling, and ultimately determines plant growth and development. In non-stressed conditions, deficiencies in Trx *h*2 and *o*1 differentially affected the reduction states of the AsA/DHA and GSH/GSSG redox couples. Trx *o*1, but not Trx *h*2, deficiency led to a decrease in both AsA (Figure 6D) and GSH reduction states (Figure 6H). The *trxo1h2* double mutant showed similar changes as the *trxo1* single mutant with no substantial additive effects. In contrast to this, the NADPH/NADP^+^ and NADH/NAD^+^ redox couples in all mutant lines were similar to the wild type, with the exception of a nocturnal increase in NADPH/NADP^+^ redox state in the *trxh2* mutant. These results indicate that, unlike Trx *h*2, Trx *o*1 is important to maintain the GSH and AsA redox states of the cell in non-stressed conditions. Since the AsA-GSH cycle mainly operates in mitochondria but has not been documented in ER [64], results are in line with the differential subcellular localization of Trxs *h*2 and *o*1, in Golgi/ER and mitochondria, respectively. This suggests that the different subcellular localization of both proteins is associated with different redox-active functions. It may also explain the differential effect of the two Trx proteins on plant growth.

From the in silico prediction, enzymes of the AsA-GSH cycle harbor at least one putative redox-reactive Cys (Table S3). In recent proteomic studies, several enzymes of the AsA-GSH cycle and NAD(P)H metabolism were found to be redox reactive, and some of them were confirmed to be Trx targets [14,65,66,67,68,69]. Interestingly, Yoshida et al. identified stromal/mitochondrial ascorbate peroxidase (At4g08390), glutathione peroxidase 6 (At4g11600), malate dehydrogenase 1 (At1g53240) and NAD^+^-dependent malic enzyme (At2g13560/At4g00570) as putative target proteins of Trx *o*1 [69]. Therefore, these proteins are the most likely candidates to be involved in the regulation of the AsA-GSH cycle and NAD(P)H metabolism by Trx *o*1. The in vivo relevance of this notion still requires further investigations.

Joint and single deficiencies of Trxs *h*2 and *o*1 also led to marked changes in nocturnal metabolite profiles, while there were no substantial changes in the profiles monitored in the light phase (Figure 4; Figure 5). The PCA of nocturnal metabolite profiles showed that all three mutant lines clustered differently from the wild type (Figure 4A). However, *trxh2* was only partly overlapping with these mutants and clusters more closely to the wild type, even though *trxo1* and *trxo1h2* clustered together. Thus, Trxs *h*2 and *o*1 partially affect global metabolite profiles in different manners, but it will not lead to additive effects when both Trxs are deficient. This further indicates that Trx *h*2 and *o*1 only share partially overlapping functions. The metabolic pathways where deficiencies of Trxs *h*2 and *o*1 led to similar patterns in metabolite profiles are as follows.

First, the levels of the TCA cycle intermediates were generally decreased in the mutants (Figure 5; Table S1). The results are in line with previous studies that proposed that Trx *o*1 and *h*2 are involved in the enzyme activation of the TCA cycle [33]. Secondly, in addition to the changes in the TCA cycle metabolites, there was an extremely strong increase of GABA in all mutant lines (Figure 5B; Table S1). Because the level of GABA precursor, Glu, was not changed in the mutants, it is likely that the accumulation of GABA is due to the degradation of polyamine, such as putrescine and spermidine [70,71]. The level of putrescine was actually decreased in all mutants (Table S1). Moreover, the over-accumulation of GABA indicates that the carbon flow of the TCA cycle in the mutants was compromised, so plants were alternatively elevating carbon flux through the GABA shunt to bypass the compromised activity of the TCA cycle enzymes [72]. Thirdly, down-regulating the metabolism of organic acids is very likely to compromise the metabolism of amino acids since organic acids serve as the major source of carbon skeleton for the biosynthesis of amino acids. As expected, the levels of many amino acids, including Gly, Ser, Gln, Met, Orn and Arg, were decreased in the mutants (Figure 5B; Table S1). Notably, the concomitant decreases of Orn and Arg indicate a possible perturbation of the Arg biosynthesis pathway [73]. In comparison to other amino acids, Arg harbors a high N:C ratio and acts as an important nitrogen storage compound. It is also a precursor for the biosynthesis of polyamines and other nitrogen-containing compounds [74,75]. Thus, perturbation in Arg biosynthesis might subsequently lead to negative effects on nitrogen metabolism. However, it is unlikely that Trx *h*2 and *o*1 are able to regulate the enzymes of the Arg biosynthesis pathway directly [73] since most of them reside in plastids. Thus, the down-regulation in Arg synthesis might be due to the shortage of organic acids. Fourthly, during the day, the levels of photorespiratory intermediates, such as Gly, Ser and glycerate, in the mutant lines were comparable to the wild type (Figure 5B; Table S1). However, it should be noted that plants were grown in normal air where the contributions of Trx *h*2 and *o*1 to photorespiration are expected to be of minor importance. Indeed, the *trxh2* and *trxo1* single mutants showed comparable carbon assimilation rates to the wild type when grown under normal air conditions [36,47]. 

Interestingly, joint deficiencies of Trx *h*2 and *o*1 had no substantial additive effects on the accumulation of most metabolites, which makes it difficult to assess how both proteins cooperate in metabolic regulation. Since Trxs *h*2 and *o*1 are located in ER/Golgi and mitochondria, respectively, interorganellar communication will be required. However, the underlying mechanisms are unresolved. While Trx *h*2 is probably anchored to the cytoplasmic face of ER/Golgi by myristoylation [38], it may affect cytosolic processes that are linked to mitochondria. Physiological connections between ER and mitochondria have also been reported in other studies where knock-out of an ATP antiporter in the ER was found to be associated with changes in ROS levels and perturbation of mitochondrial steps of photorespiration [76]. In addition to this, interorganellar communication will be facilitated by physical membrane contact between ER and mitochondria, which has been documented recently by genetically encoded reporters [77]. More work will be required to elucidate ER-mitochondria interactions in the regulation of plant metabolism. 

### 4.3. Thioredoxin h2 and o1 Jointly Affect Photosynthetic Efficiency in Fluctuating Light

Plants have to manage strong light fluctuations in the field. Rapid alterations in light intensity strongly affect the availability of light energy for photosynthetic electron transport and carbon fixation and require efficient acclimation mechanisms to maintain photosynthetic efficiency and plant growth [1,2]. It has been found that light-dependent chloroplast Trxs play a crucial role in the dynamic acclimation of photosynthesis in fluctuating light [19]. Our results show that this extends also to NADPH-dependent extra-plastidial Trxs. Intriguingly, the *trxo1h2* double mutant revealed a higher photosynthetic efficiency than the wild type and single mutants under fluctuating light, especially in the HL phases (Figure 8). This indicates that the extra-chloroplastidial Trxs *h*2 and *o*1 act cooperatively to dampen acclimation processes in fluctuating light.

Acclimation processes in fluctuating light were proposed to be important to avoid imbalances in NADPH/NADP^+^ redox states. However, studies are largely lacking to test this notion. Our results confirm the vulnerability of the NADPH/NADP^+^ redox state in fluctuating light conditions. In the wild type, there is a strong decline in the NADPH/NADP^+^ redox state in fluctuating light, compared to constant ML, which is quite dramatic since it drops down below nocturnal levels (Figure 7C). Interestingly, in the *trxo1h2* double mutant, the FL-dependent decrease in the NADPH/NADP^+^ ratio was attenuated, specifically in the LL phase of FL (Figure 7C), which is in line with its improved photosynthetic efficiency under these conditions. This indicates that the cooperative effect of extra-plastidial Trxs *h*2 and *o*1 on FL-dependent acclimation processes is linked to NADPH redox homeostasis. The underlying mechanisms, however, remain unresolved. At the moment, it is unclear how Trxs *h*2 and *o*1, which are associated with Golgi/ER and mitochondria, respectively, can affect FL-acclimation processes that are known to reside in the chloroplast [78]. The cellular NADPH homeostasis is known to be mediated by inter-organellar malate/oxaloacetate shuttles involving the activities of different malate dehydrogenase (MDH) proteins distributed to chloroplasts, mitochondria, peroxisomes and cytoplasm [79]. Proteomics studies suggested that MDH proteins might act as targets of *h*-type Trxs [66,67]. Specifically, the activity of mitochondrial NAD^+^-dependent MDH is proposed to be regulated by Trx *o*1 [33]. Since Trx *h*2 is probably anchored to the cytoplasmic face of ER/Golgi by myristoylation [38], it may regulate cytoplasmic MDH activity [80]. Taken together, it is likely that Trx *h*2 and *o*1 might cooperatively modulate the activities of MDH isoforms and, thus, affect the cellular redox balance of NADPH and finally photosynthetic efficiency in FL.

Acclimation in fluctuating light has also been proposed to involve stimulation of photorespiratory processes [81,82,83], providing an alternative explanation for the cooperative role of Trxs *h*2 and *o*1 in this context. Deficiencies of Trx *h*2 and *o*1 have been found to enhance the activity of glycine decarboxylation (GDC) in mitochondria. This would produce high amounts of NADH and further facilitate the operation of mitochondrial electron transport chain and photorespiratory carbon flow [36,47]. Enhancing photorespiratory carbon flow is proposed to promote photosynthesis [84,85]. It is quite likely that this mechanism is specifically important in FL conditions, which require elevated photorespiratory capacities [81,82,83]. Indeed, single and joint deficiencies of Trx *h*2 and *o*1 affected Gly level in FL conditions, leading to an increase in the LL phase, while there was a decrease in the HL phase of FL (Figure 5E). This suggests that Trxs *h*2 and *o*1 proteins are operating as negative effectors of FL acclimation since they negatively regulate photorespiratory capacity under these conditions. Nevertheless, it must be noted that over accumulation of NADH in mitochondria strongly suppresses GDC activity and decreases the photorespiratory flow [86]. This might eventually compromise the performance of photosynthesis. Thus, the operation described above might only occur when plants undergo a short-term fluctuation of different light intensities. 

Further investigations are required to confirm the two hypotheses mentioned above and to decipher the underlying regulatory mechanisms. The physiological connections between ER, mitochondria and chloroplasts may also be facilitated by direct membrane contact sites [77].

## 5. Conclusions

This study finally clarifies the subcellular localization of Trx *h*2, providing direct biochemical evidence that Trx *h*2 proteins are associated to microsomes rather than to mitochondria, documenting a different localization in comparison to Trx *o*1. The different subcellular localization of both proteins is associated with different redox-active functions, with Trx *o*1, but not Trx *h*2, being important for maintaining the AsA and GSH redox states and plant growth in non-stressed conditions. In contrast to this, there might be a cooperative role of both Trxs *o*1 and *h*2 in regulating NADPH redox balance and photosynthetic performance in fluctuating light environments. This suggests a possible physiological interaction of both proteins between ER/Golgi and mitochondria, which extends to photosynthetic acclimation in the chloroplast.

## Data Availability

Data is contained within the article or Supplementary Materials.

## Supplementary Materials

The following are available online at https://www.mdpi.com/article/10.3390/antiox10050705/s1, Figure S1: Molecular characterization of Trx *h*2 knock-out mutant (*trxh2*) and over expression line (Trx *h*2_ox_). Figure S2: The quantum yield of photosystem II (Y(II)) in the wild type (Col-0) and the mutant lines (*trxh2*, *trxo1* and *trxo1h2*) in medium light. Table S1: Fold changes in metabolite profiles in Arabidopsis leaves of the wild type (Col-0) and thioredoxin mutant lines (*trxh2*, *trxo1* and *trxo1h2*) grown in non-stressed medium-light conditions. Table S2: Fold changes in metabolite profiles in Arabidopsis leaves of the wild type (Col-0) and thioredoxin mutant lines (*trxh2*, *trxo1* and *trxo1h2*) grown in fluctuating light conditions. Table S3: Prediction of conserved cysteine in enzymes of AsA-GSH cycle.

## Material Distribution Statement

The author responsible for distribution of materials integral to the findings presented in this article in accordance with the policy described in the Instructions for Authors is: Peter Geigenberger (geigenberger@bio.lmu.de).
