# Supplementary material for: Thioredoxin h2 and o1 Show Different Subcellular Localizations and Redox-Active Functions, and Are Extrachloroplastic Factors Influencing Photosynthetic Performance in Fluctuating Light
Source: Antioxidants (Basel). 2021 Apr 29;10(5):705. doi: 10.3390/antiox10050705 (PMC8147087; doi:10.3390/antiox10050705)
Supplement: Supplementary file 1 [file antioxidants-10-00705-s001.zip › antioxidants-1177726-supplementary.pdf]

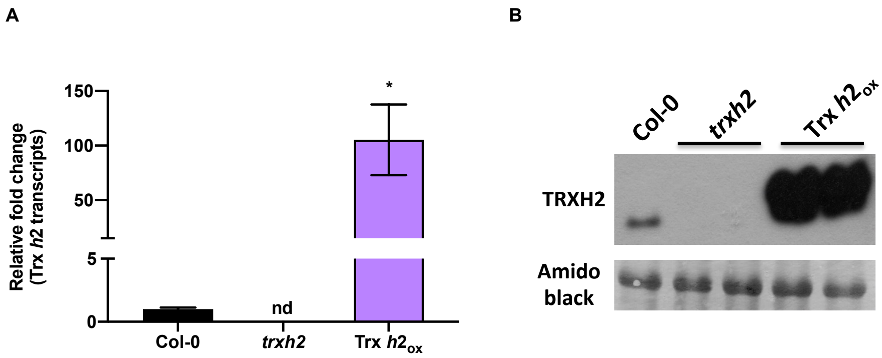

Figure S1. Molecular characterization of Trx *h*2 knock-out mutant (*trxh2*) and over expression line (Trx $h2_{ox}$). A, The transcript level of Trx *h*2 mutant and overexpression line compared to the wild type (Col-0). B, The protein level of Trx *h*2 mutant and overexpression line compared to the wild type (Col-0). The total protein visualized by amino black staining serves as loading control. Mean values and standard errors derived from 3 biological replicates. The statistical analyses were performed by using ANOVA and the Dunnett's test (*P < 0.05, in comparison to the wild type).

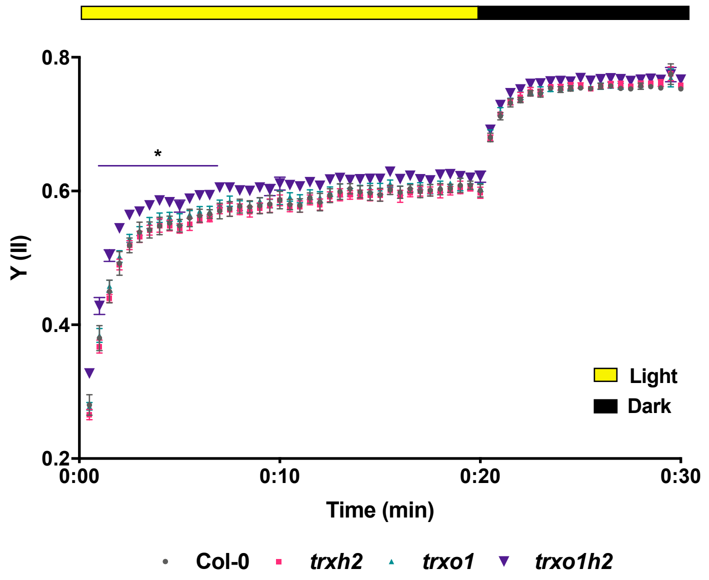

Figure S2. The quantum yield of photosystem II [Y(II)] in the wild type (Col-0) and the mutant lines (*trxh2*, *trxo1* and *trxo1h2*) in medium light. The Arabidopsis plants were grown in a medium light environment for three weeks. Chlorophyll florescence kinetics during the period of medium light were recorded by using a PAM system, and the values were used for calculating Y(II). Mean values and standard errors derived from 6 biological replicates. The statistical analyses were performed by using ANOVA and the Dunnett's test (*$P < 0.05$, in comparison to the wild type).

Table S1. Fold changes in metabolite profiles in Arabidopsis leaves of the wild type (Col-0) and thioredoxin mutant lines (*trxh2*, *trxo1* and *trxo1h2*) grown in non-stressed medium-light conditions. Arabidopsis rosette leaves were harvested at the end of night (EN) and the end of day (ED). Mean values and standard errors derived from 6 biological replicates. The statistical analyses were performed by using ANOVA and the Dunnett's test (*P < 0.05, in comparison to the wild type).

**Time point**	**Arabidopsis leaves (EN)**						
**Genotype**	**Col-0**	***trxh2***		***trxo1***		***trxo1h2***	
**Metabolite**	**Fold change**	**Fold change**	**p-value**	**Fold change**	**p-value**	**Fold change**	**p-value**
**Soluble sugars**							
Arabinose	1.00±0.02	1.10±0.03	0.7130	0.97±0.07	0.9820	1.03±0.14	0.9880
Erythrose	1.00±0.11	1.39±0.14	0.6458	2.39±0.35	**0.0058**	1.84±0.39	0.1142
Fructose	1.00±0.30	0.69±0.17	0.5090	0.84±0.11	0.8740	0.51±0.03	0.1680
Fucose	1.00±0.08	0.95±0.08	0.9372	0.88±0.08	0.5190	0.73±0.06	0.0517
Glucoheptose	1.00±0.06	0.88±0.06	0.2250	0.64±0.03	**0.0010**	0.65±0.04	**0.0010**
Glucose	1.00±0.06	1.02±0.08	0.9930	1.19±0.12	0.2450	1.14±0.05	0.4990
2-deoxy-glucose	1.00±0.07	0.72±0.11	0.3070	1.16±0.12	0.6950	1.23±0.19	0.4590
Maltose	1.00±0.04	1.05±0.06	0.8270	1.04±0.04	0.9180	0.94±0.06	0.7430
Mannose	1.00±0.13	1.22±0.10	0.4540	1.22±0.13	0.4500	1.28±0.12	0.2650
Psicose	1.00±0.19	0.97±0.08	0.9970	1.01±0.06	1.0000	0.97±0.09	0.9950
Raffinose	1.00±0.19	0.54±0.11	**0.0231**	0.54±0.06	**0.0218**	0.37±0.03	**0.0020**
Sucrose	1.00±0.05	1.02±0.07	0.9760	0.87±0.03	0.1640	0.95±0.04	0.8480
Threose	1.00±0.08	0.96±0.05	0.9937	1.51±0.19	0.0733	1.29±0.22	0.4211
Trehalose	1.00±0.08	1.04±0.02	0.8950	0.90±0.05	0.4920	0.90±0.05	0.4580
Xylose	1.00±0.04	1.08±0.06	0.6510	0.98±0.05	0.9870	1.02±0.07	0.9930
Xylulose	1.00±0.07	0.83±0.04	0.3510	0.87±0.06	0.5700	0.81±0.12	0.2740
**Sugar alcohols**							
Arabitol	1.00±0.11	0.86±0.13	0.6350	0.80±0.05	0.3590	0.79±0.10	0.3380
Galactinol	1.00±0.09	0.98±0.02	0.9850	0.86±0.04	0.2090	0.89±0.05	0.3920
Glycerol	1.00±0.02	1.15±0.10	0.5680	1.20±0.13	0.3290	0.93±0.10	0.9150
Glycerophosphoglycerol	1.00±0.11	0.93±0.04	0.8159	0.81±0.03	0.1483	0.75±0.05	**0.0406**
Myo-Inositol	1.00±0.10	0.97±0.06	0.9789	0.77±0.05	0.0654	0.78±0.06	0.0895
Threitol	1.00±0.08	0.73±0.11	0.1104	0.85±0.09	0.4991	0.63±0.07	**0.0192**
Xylitol	1.00±0.08	0.87±0.11	0.7020	0.84±0.07	0.5680	0.79±0.14	0.3350

**Organic acids**							
2-piperidinecarboxylic acid	1.00±0.24	0.54±0.09	0.0531	0.36±0.04	**0.0068**	0.30±0.04	**0.0031**
Adipic acid	1.00±0.16	0.71±0.07	0.1550	0.57±0.09	**0.0268**	0.56±0.08	**0.0215**
Arabinonic acid	1.00±0.15	1.05±0.11	0.9970	1.38±0.28	0.5750	1.39±0.37	0.5680
Ascorbic acid	1.00±0.14	0.83±0.02	0.2580	0.29±0.04	**0.0010**	0.39±0.02	**0.0010**
Aspartic acid	1.00±0.05	0.90±0.06	0.6380	0.90±0.08	0.5893	0.76±0.08	0.0678
Azelaic Acid	1.00±0.08	0.71±0.11	0.2020	1.03±0.10	0.9950	1.09±0.14	0.9040
Benzoic acid	1.00±0.05	1.13±0.07	0.6260	1.10±0.11	0.7620	0.98±0.11	0.9970
4-hydroxybenzoic acid	1.00±0.20	1.25±0.18	0.5480	1.31±0.14	0.3900	1.23±0.10	0.6230
2,4-dihydroxybutanoic acid	1.00±0.11	1.30±0.15	0.3630	1.17±0.16	0.7570	1.28±0.15	0.4140
4-acetamidobutanoic acid	1.00±0.04	0.88±0.07	0.4485	0.88±0.08	0.4759	0.74±0.08	**0.0362**
Dehydroascorbic acid	1.00±0.16	0.90±0.06	0.8400	1.05±0.07	0.9710	0.97±0.12	0.9960
Erythronic acid	1.00±0.12	1.02±0.14	0.9990	0.93±0.10	0.9570	0.97±0.13	0.9980
Galactaric acid	1.00±0.10	1.08±0.07	0.9120	1.29±0.08	0.1280	1.07±0.14	0.9370
Galactonic acid	1.00±0.08	1.01±0.05	1.0000	0.88±0.01	0.4000	0.84±0.08	0.2000
Glucoheptonic acid	1.00±0.07	0.96±0.09	0.9670	0.82±0.04	0.2070	0.88±0.07	0.5130
Gluconic acid	1.00±0.03	0.88±0.03	0.1769	0.75±0.02	**0.0022**	0.68±0.08	**0.0010**
Gluconic acid-1,4-lactone	1.00±0.03	1.05±0.10	0.9780	1.30±0.16	0.1390	1.18±0.09	0.5050
3-oxoglutaric acid	1.00±0.12	0.83±0.08	0.4244	0.78±0.05	0.2285	0.65±0.09	**0.0305**
Glyceric acid	1.00±0.09	0.90±0.13	0.9210	0.90±0.14	0.9100	0.96±0.17	0.9920
Iminodiacetic acid	1.00±0.09	1.54±0.18	0.2294	2.61±0.34	**0.0010**	2.03±0.20	**0.0098**
Lactic acid	1.00±0.08	0.61±0.05	**0.0010**	0.55±0.03	**0.0010**	0.69±0.06	**0.0023**
Nicotinic acid	1.00±0.08	0.86±0.06	0.4590	0.80±0.06	0.2200	0.87±0.10	0.5100
6-hydroxynicotinic acid	1.00±0.12	0.97±0.10	0.9960	0.78±0.11	0.3560	1.08±0.08	0.9180
Phosphoric acid	1.00±0.07	1.26±0.11	0.1540	1.03±0.11	0.9900	0.86±0.08	0.6090
Ribonic acid	1.00±0.12	0.76±0.10	0.2001	0.69±0.07	0.0771	0.57±0.07	**0.0105**
Shikimic acid	1.00±0.10	0.72±0.10	0.0862	0.76±0.08	0.1527	0.84±0.07	0.4506
Cis-sinapic acid	1.00±0.06	0.85±0.04	0.3288	0.75±0.08	0.0575	0.71±0.09	**0.0258**
Threonic acid	1.00±0.11	1.12±0.09	0.8120	1.18±0.13	0.5810	1.23±0.14	0.3850
Uric acid	1.00±0.05	1.01±0.08	0.9980	0.91±0.11	0.7670	1.08±0.06	0.7830
1-pyrroline-2-carboxylate	1.00±0.11	1.24±0.20	0.7360	1.25±0.28	0.6950	0.66±0.14	0.4890
**Amino acids**							
3-iodotyrosine	1.00±0.07	0.97±0.06	0.9700	0.88±0.04	0.3520	0.78±0.06	**0.0350**
4-aminobutanoic acid	1.00±0.10	1.70±0.20	0.1688	2.93±0.39	**0.0010**	2.24±0.27	**0.0084**

Alanine	1.00±0.05	1.00±0.06	1.0000	1.01±0.05	0.9900	1.01±0.09	0.9900
Arginine	1.00±0.08	0.76±0.11	0.2400	0.84±0.10	0.5450	0.79±0.10	0.3600
Asparagine	1.00±0.06	0.89±0.04	0.6740	1.00±0.10	1.0000	0.85±0.11	0.4480
Beta- alanine	1.00±0.07	1.40±0.15	0.1680	1.01±0.13	1.0000	1.42±0.22	0.1460
DL-2-aminobutyric acid	1.00±0.05	1.10±0.07	0.8520	1.01±0.13	1.0000	1.28±0.16	0.2020
DL-glutamine	1.00±0.12	0.73±0.06	0.0838	0.88±0.06	0.6089	0.71±0.08	0.0610
Glutamic acid	1.00±0.09	1.08±0.07	0.7160	0.98±0.05	0.9950	0.91±0.05	0.6660
Glycine	1.00±0.06	0.78±0.08	0.2034	0.61±0.07	**0.0123**	0.75±0.12	0.1339
Glycylglycylglycine	1.00±0.10	0.96±0.05	0.9450	1.02±0.05	0.9960	0.87±0.07	0.4520
Homoserine	1.00±0.09	1.06±0.07	0.9660	1.11±0.11	0.8290	0.92±0.17	0.9160
Isoleucine	1.00±0.07	0.97±0.08	0.9930	0.93±0.12	0.9310	0.89±0.13	0.7920
Leucine	1.00±0.07	1.27±0.10	0.1750	1.13±0.13	0.7000	0.96±0.10	0.9850
Methionine	1.00±0.14	0.83±0.11	0.5650	0.83±0.09	0.5670	0.84±0.10	0.6110
O-acetylserine	1.00±0.08	1.27±0.18	0.3490	0.93±0.11	0.9670	1.06±0.15	0.9750
Ornithine	1.00±0.11	0.80±0.09	0.2740	0.76±0.07	0.1480	0.63±0.07	**0.0170**
Ornithine-1,5-lactam	1.00±0.15	0.92±0.09	0.9730	1.40±0.13	0.1940	1.01±0.23	1.0000
Phenylalanine	1.00±0.10	1.40±0.12	**0.0394**	1.31±0.09	0.1229	1.36±0.11	0.0701
Proline	1.00±0.05	1.02±0.07	0.9970	0.93±0.12	0.9300	0.87±0.14	0.6640
Pyroglutamic acid	1.00±0.07	0.88±0.06	0.5960	0.85±0.09	0.4640	0.86±0.11	0.5180
Serine	1.00±0.14	0.89±0.11	0.8230	0.73±0.07	0.2390	0.84±0.12	0.6120
Threonine	1.00±0.08	1.14±0.11	0.6360	1.02±0.09	0.9970	1.06±0.12	0.9560
Trans-4-hydroxyproline	1.00±0.11	0.81±0.10	0.4100	0.79±0.09	0.3260	0.80±0.10	0.3690
Tryptophan	1.00±0.11	1.04±0.09	0.9810	0.88±0.07	0.7200	1.08±0.13	0.8980
Valine	1.00±0.06	1.08±0.10	0.9230	1.04±0.10	0.9820	1.21±0.15	0.3840
**TCA cycle intermediates**							
Cis-aconitic acid	1.00±0.08	0.92±0.08	0.8586	0.90±0.12	0.7614	0.64±0.08	**0.0301**
Citric acid	1.00±0.05	1.21±0.10	0.1580	1.20±0.09	0.1640	1.11±0.04	0.5850
Fumaric acid	1.00±0.11	1.01±0.18	1.0000	0.80±0.03	0.4400	0.84±0.04	0.6000
2-oxoglutaric acid	1.00±0.04	0.82±0.11	0.3097	0.69±0.10	**0.0345**	0.71±0.05	0.0519
Malic acid	1.00±0.12	1.08±0.21	0.9440	1.03±0.06	0.9960	0.93±0.05	0.9530
Pyruvic acid	1.00±0.03	0.82±0.06	0.0700	0.71±0.04	**0.0031**	0.78±0.07	**0.0213**
Succinic acid	1.00±0.08	0.81±0.10	0.3388	0.67±0.08	0.0555	0.72±0.11	0.1033
2-methylmalic acid	1.00±0.12	0.92±0.19	0.9610	1.01±0.09	1.0000	0.76±0.13	0.4850
2,3-dimethylsuccinic acid	1.00±0.14	0.84±0.08	0.6070	0.70±0.10	0.1480	0.74±0.10	0.2300

**Phosphate intermediates**							
Fructose-6-phosphate	1.00±0.07	0.67±0.08	**0.0064**	0.82±0.05	0.1613	0.74±0.05	**0.0312**
Glucose-6-phosphate	1.00±0.08	0.98±0.07	0.9860	0.85±0.04	0.2460	0.83±0.05	0.1520
2-amino-2-deoxyglucose-6-phosphate	1.00±0.15	1.02±0.07	0.9980	0.69±0.06	0.0853	0.75±0.08	0.1932
Glycerol-3-phosphate	1.00±0.11	0.87±0.06	0.5420	0.74±0.08	0.0868	0.59±0.07	**0.0064**
**Miscellaneous**							
5-methylthioadenosine	1.00±0.08	1.04±0.04	0.9600	0.99±0.02	0.9990	1.13±0.08	0.3670
Butylamine	1.00±0.09	0.83±0.04	0.1756	0.90±0.04	0.5833	0.75±0.07	**0.0313**
Putrescine	1.00±0.09	0.73±0.06	**0.0302**	0.64±0.06	**0.0045**	0.62±0.06	**0.0030**
2,3-dihydroxypyridine	1.00±0.06	0.68±0.09	**0.0189**	0.47±0.05	**0.0010**	0.54±0.09	**0.0010**
Spermidine	1.00±0.10	1.16±0.13	0.6770	0.98±0.08	0.9990	1.23±0.15	0.3870
Sphingosine	1.00±0.08	0.87±0.08	0.3920	0.86±0.04	0.3360	0.82±0.05	0.1850
Ethanolamine	1.00±0.10	1.60±0.13	0.1237	2.16±0.29	**0.0018**	1.80±0.24	**0.0310**
1,2,4-triolbenzene	1.00±0.31	0.89±0.16	0.9600	0.71±0.07	0.6180	0.84±0.18	0.8950
Nicotinamide	1.00±0.08	0.81±0.12	0.4072	0.81±0.09	0.4077	0.69±0.09	0.0848
Uracil	1.00±0.07	0.88±0.11	0.6850	0.76±0.07	0.1880	0.83±0.11	0.4130
Urea	1.00±0.08	0.84±0.07	0.3350	0.75±0.06	0.0740	0.82±0.09	0.2360
3-indoleacetonitrile	1.00±0.05	1.11±0.10	0.6450	0.97±0.09	0.9840	0.99±0.08	0.9990

Time point	Arabidopsis leaves (ED)						
Genotype	Col-0	*trxh2*		*trxo1*		*trxo1h2*	
Metabolite	Fold change	Fold change	p-value	Fold change	p-value	Fold change	p-value
**Soluble sugars**							
Arabinose	1.00±0.07	1.02±0.11	0.9980	0.87±0.06	0.5400	0.95±0.08	0.9370
Erythrose	1.00±0.12	0.83±0.17	0.7110	0.79±0.10	0.5780	0.83±0.13	0.7150
Fructose	1.00±0.25	0.73±0.05	0.4000	0.89±0.05	0.9040	1.12±0.07	0.8710
Fucose	1.00±0.09	1.18±0.12	0.3660	1.01±0.07	1.0000	1.01±0.07	1.0000
Glucoheptose	1.00±0.07	1.50±0.18	**0.0167**	1.17±0.08	0.5908	1.18±0.10	0.5487
Glucose	1.00±0.24	1.03±0.10	0.9980	1.06±0.08	0.9850	1.22±0.08	0.5680
2-deoxy-glucose	1.00±0.29	1.69±0.48	0.3560	1.36±0.25	0.7950	1.40±0.28	0.7340
Maltose	1.00±0.36	2.28±0.28	0.5682	3.91±0.76	0.0554	13.77±1.40	**0.0010**
Mannose	1.00±0.08	1.09±0.09	0.7570	0.97±0.06	0.9800	1.12±0.08	0.5740
Psicose	1.00±0.08	1.08±0.07	0.7900	1.00±0.09	1.0000	1.01±0.07	0.9990
Raffinose	1.00±0.23	1.55±0.21	0.0778	1.21±0.08	0.7197	1.13±0.08	0.9048
Sucrose	1.00±0.09	0.90±0.02	0.5910	1.06±0.05	0.8390	1.15±0.08	0.2430
Threose	1.00±0.08	0.88±0.13	0.7010	0.90±0.07	0.8140	0.88±0.09	0.7280
Trehalose	1.00±0.10	1.13±0.15	0.6810	1.05±0.06	0.9660	1.25±0.06	0.2110
Xylose	1.00±0.07	1.11±0.10	0.6390	1.00±0.06	1.0000	1.01±0.06	1.0000
Xylulose	1.00±0.09	0.94±0.11	0.9320	0.85±0.08	0.5600	0.94±0.10	0.9460
**Sugar alcohols**							
Arabitol	1.00±0.11	1.29±0.18	0.2750	1.14±0.10	0.7800	1.09±0.11	0.9150
Galactinol	1.00±0.08	1.03±0.13	0.9890	0.97±0.06	0.9870	0.94±0.06	0.9400
Glycerol	1.00±0.12	0.86±0.15	0.7930	1.01±0.16	1.0000	0.89±0.07	0.8690
Glycerophosphoglycerol	1.00±0.08	1.14±0.12	0.4950	0.96±0.06	0.9680	1.07±0.06	0.8710
Myo-Inositol	1.00±0.08	1.08±0.09	0.7980	1.11±0.07	0.6000	1.05±0.05	0.9500
Threitol	1.00±0.09	1.09±0.12	0.8540	0.96±0.08	0.9790	0.96±0.08	0.9820
Xylitol	1.00±0.05	1.01±0.11	0.9990	0.97±0.07	0.9870	0.97±0.08	0.9860
**Organic acids**							
2-piperidinecarboxylic acid	1.00±0.41	0.44±0.09	0.1900	0.52±0.08	0.2870	0.56±0.07	0.3590
Adipic acid	1.00±0.09	1.04±0.13	0.9860	0.93±0.08	0.9120	0.85±0.07	0.5320
Arabinonic acid	1.00±0.24	0.82±0.27	0.8760	0.53±0.14	0.3000	0.57±0.18	0.3750
Ascorbic acid	1.00±0.16	1.17±0.11	0.7490	0.94±0.16	0.9800	0.64±0.15	0.2190

Aspartic acid	1.00±0.11	1.07±0.17	0.9630	0.94±0.11	0.9720	0.95±0.07	0.9780
Azelaic Acid	1.00±0.18	1.52±0.36	0.3600	1.34±0.22	0.6610	1.30±0.21	0.7360
Benzoic acid	1.00±0.23	1.08±0.21	0.9770	0.79±0.10	0.7130	0.73±0.07	0.5440
4-hydroxybenzoic acid	1.00±0.15	1.12±0.23	0.9480	1.00±0.21	1.0000	0.91±0.14	0.9740
2,4-dihydroxybutanoic acid	1.00±0.14	0.98±0.15	0.9980	0.85±0.08	0.7180	0.85±0.11	0.7490
4-acetamidobutanoic acid	1.00±0.10	1.05±0.16	0.9820	0.96±0.12	0.9880	0.95±0.06	0.9810
Dehydroascorbic acid	1.00±0.12	1.49±0.30	0.2430	1.27±0.20	0.6860	1.24±0.15	0.7540
Erythronic acid	1.00±0.09	1.16±0.10	0.4150	0.94±0.07	0.9080	0.95±0.07	0.9410
Galactaric acid	1.00±0.08	1.34±0.28	0.3650	1.19±0.14	0.7680	1.01±0.08	1.0000
Galactonic acid	1.00±0.09	1.13±0.16	0.6820	1.05±0.08	0.9710	0.95±0.06	0.9710
Glucoheptonic acid	1.00±0.07	1.02±0.08	0.9960	0.89±0.06	0.4950	0.97±0.05	0.9720
Gluconic acid	1.00±0.09	0.98±0.08	0.9940	0.97±0.08	0.9860	1.00±0.09	1.0000
Gluconic acid-1,4-lactone	1.00±0.12	1.20±0.07	0.4990	1.18±0.13	0.5570	1.31±0.14	0.1800
3-oxoglutaric acid	1.00±0.29	2.50±0.91	0.1650	1.60±0.48	0.7860	1.37±0.27	0.9330
Glyceric acid	1.00±0.07	1.02±0.11	0.9980	0.93±0.12	0.9330	0.75±0.07	0.1870
Iminodiacetic acid	1.00±0.17	1.17±0.15	0.7310	1.31±0.07	0.3110	2.27±0.15	**0.0010**
Lactic acid	1.00±0.13	1.01±0.19	1.0000	1.10±0.21	0.9410	0.93±0.07	0.9820
Nicotinic acid	1.00±0.07	1.15±0.15	0.6820	1.09±0.11	0.9040	1.02±0.12	0.9990
6-hydroxynicotinic acid	1.00±0.11	1.09±0.15	0.8850	1.01±0.08	1.0000	1.05±0.09	0.9830
Phosphoric acid	1.00±0.14	1.30±0.34	0.7280	1.10±0.31	0.9830	0.83±0.11	0.9220
Ribonic acid	1.00±0.13	1.16±0.13	0.6250	0.80±0.08	0.4700	0.85±0.11	0.6750
Shikimic acid	1.00±0.08	1.02±0.11	0.9940	0.94±0.07	0.9050	0.92±0.07	0.8420
Cis-sinapic acid	1.00±0.10	1.27±0.23	0.5220	1.06±0.16	0.9890	1.03±0.12	0.9980
Threonic acid	1.00±0.11	1.11±0.13	0.7470	0.94±0.06	0.9540	0.90±0.07	0.7940
Uric acid	1.00±0.11	1.05±0.09	0.9580	1.00±0.09	1.0000	1.08±0.08	0.8840
1-pyrroline-2-carboxylate	1.00±0.36	0.69±0.10	0.7120	0.93±0.29	0.9950	0.85±0.15	0.9520
**Amino acids**							
3-iodotyrosine	1.00±0.09	0.81±0.05	0.1400	0.85±0.06	0.2750	0.89±0.06	0.5190
4-aminobutanoic acid	1.00±0.44	2.16±0.32	**0.0483**	2.44±0.08	**0.0130**	4.90±0.33	**0.0010**
Alanine	1.00±0.15	1.30±0.15	0.3538	1.80±0.12	**0.0027**	2.41±0.16	**0.0010**
Arginine	1.00±0.12	0.75±0.11	0.2070	0.81±0.08	0.4020	0.81±0.08	0.4090
Asparagine	1.00±0.14	1.05±0.19	0.9840	1.00±0.09	1.0000	1.07±0.10	0.9710
Beta- alanine	1.00±0.07	1.37±0.25	0.2140	1.37±0.14	0.2220	1.18±0.05	0.7130
DL-2-aminobutyric acid	1.00±0.07	1.44±0.34	0.2570	0.98±0.10	1.0000	0.95±0.09	0.9960

DL-glutamine	1.00±0.11	0.98±0.10	0.9990	0.91±0.08	0.8370	1.00±0.09	1.0000
Glutamic acid	1.00±0.10	1.07±0.15	0.9290	0.97±0.08	0.9920	0.99±0.07	1.0000
Glycine	1.00±0.21	0.99±0.19	1.0000	1.31±0.22	0.5540	0.88±0.15	0.9480
Glycylglycylglycine	1.00±0.07	0.81±0.07	0.2080	0.83±0.07	0.2710	0.94±0.09	0.8800
Homoserine	1.00±0.09	0.73±0.11	0.0820	0.74±0.07	0.1081	0.59±0.07	**0.0079**
Isoleucine	1.00±0.15	0.84±0.09	0.9270	1.20±0.37	0.8730	1.05±0.19	0.9980
Leucine	1.00±0.15	0.80±0.14	0.6730	1.12±0.21	0.8990	0.92±0.07	0.9660
Methionine	1.00±0.09	1.00±0.12	1.0000	0.96±0.06	0.9740	0.90±0.07	0.7660
O-acetylserine	1.00±0.14	1.44±0.15	**0.0478**	1.32±0.06	0.1756	2.06±0.11	**0.0010**
Ornithine	1.00±0.18	0.96±0.17	0.9970	0.70±0.10	0.3870	0.72±0.14	0.4410
Ornithine-1,5-lactam	1.00±0.10	0.77±0.15	0.3040	0.73±0.06	0.2100	0.77±0.10	0.3330
Phenylalanine	1.00±0.10	0.90±0.11	0.7610	0.89±0.07	0.7140	0.91±0.08	0.8440
Proline	1.00±0.13	0.96±0.11	0.9980	1.32±0.26	0.5640	1.38±0.26	0.4370
Pyroglutamic acid	1.00±0.14	0.87±0.12	0.7490	0.98±0.08	0.9990	0.93±0.11	0.9530
Serine	1.00±0.08	1.13±0.15	0.7630	1.07±0.13	0.9500	1.01±0.08	1.0000
Threonine	1.00±0.06	0.97±0.09	0.9910	1.07±0.12	0.8980	0.97±0.05	0.9880
Trans-4-hydroxyproline	1.00±0.10	1.02±0.14	0.9990	0.99±0.07	0.9990	0.98±0.09	0.9990
Tryptophan	1.00±0.19	1.17±0.32	0.9290	1.12±0.19	0.9680	0.89±0.24	0.9750
Valine	1.00±0.19	1.14±0.18	0.9210	1.25±0.23	0.6990	1.23±0.18	0.7420
**TCA cycle intermediates**							
Cis-aconitic acid	1.00±0.15	0.98±0.14	0.9990	0.85±0.15	0.7860	0.87±0.10	0.8310
Citric acid	1.00±0.06	0.87±0.07	0.4060	0.92±0.09	0.7090	0.86±0.04	0.3280
Fumaric acid	1.00±0.08	1.38±0.08	**0.0021**	1.05±0.07	0.9296	0.98±0.04	0.9964
2-oxoglutaric acid	1.00±0.13	0.85±0.12	0.6530	0.69±0.08	0.1340	0.71±0.09	0.1720
Malic acid	1.00±0.08	0.92±0.04	0.7920	0.97±0.11	0.9850	0.90±0.05	0.6860
Pyruvic acid	1.00±0.14	0.98±0.13	0.9980	1.02±0.16	1.0000	0.83±0.07	0.6630
Succinic acid	1.00±0.16	1.36±0.21	0.2410	1.00±0.11	1.0000	1.09±0.07	0.9470
2-methylmalic acid	1.00±0.09	1.11±0.10	0.6790	0.94±0.09	0.9400	0.90±0.06	0.7460
2,3-dimethylsuccinic acid	1.00±0.10	1.17±0.10	0.3350	0.99±0.06	1.0000	0.99±0.05	1.0000
**Phosphate intermediates**							
Fructose-6-phosphate	1.00±0.14	0.86±0.11	0.7890	1.05±0.15	0.9860	0.93±0.12	0.9650
Glucose-6-phosphate	1.00±0.12	1.34±0.21	0.1793	1.01±0.08	0.9997	1.40±0.07	0.0997
2-amino-2-deoxyglucose-6-phosphate	1.00±0.16	1.28±0.12	0.2230	1.08±0.08	0.9350	1.26±0.08	0.2650
Glycerol-3-phosphate	1.00±0.12	1.24±0.19	0.4150	0.97±0.09	0.9980	1.02±0.08	0.9980

**Miscellaneous**							
5-methylthioadenosine	1.00±0.06	1.11±0.10	0.6230	1.05±0.07	0.9290	1.08±0.06	0.7800
Butylamine	1.00±0.06	1.32±0.34	0.5140	1.01±0.14	1.0000	0.95±0.08	0.9950
Putrescine	1.00±0.07	0.96±0.12	0.9730	0.93±0.07	0.8620	0.88±0.04	0.5840
2,3-dihydroxypyridine	1.00±0.09	1.36±0.20	0.1700	1.00±0.14	1.0000	0.91±0.06	0.9310
Spermidine	1.00±0.06	1.07±0.17	0.9500	0.99±0.10	1.0000	0.92±0.07	0.9150
Sphingosine	1.00±0.08	1.02±0.10	0.9980	0.97±0.10	0.9860	0.90±0.06	0.7210
Ethanolamine	1.00±0.14	0.69±0.14	0.3300	0.94±0.15	0.9800	1.00±0.15	1.0000
1,2,4-triolbenzene	1.00±0.13	1.32±0.30	0.5080	1.03±0.12	0.9990	1.04±0.15	0.9980
Nicotinamide	1.00±0.08	1.10±0.11	0.7560	0.98±0.09	0.9980	0.99±0.08	0.9990
Uracil	1.00±0.14	1.35±0.35	0.5360	0.96±0.15	0.9990	0.92±0.14	0.9880
Urea	1.00±0.16	1.46±0.28	0.2170	0.79±0.07	0.7680	1.04±0.19	0.9980
3-indoleacetonitrile	1.00±0.09	1.01±0.13	0.9990	0.98±0.07	0.9980	1.02±0.07	0.9960

Table S2. Fold changes in metabolite profiles in Arabidopsis leaves of the wild type (Col-0) and thioredoxin mutant lines (*trxh2*, *trxo1* and *trxo1h2*) grown in fluctuating light conditions. Arabidopsis rosette leaves were harvested at the high-light (HL) and low-light (LL) phases. Mean values and standard errors derived from 5 to 6 biological replicates. The statistical analyses were performed by using ANOVA and the Dunnett's test (*P < 0.05, in comparison to the wild type).

**Phase**	**Arabidopsis leaves (HL)**						
**Genotype**	**Col-0**	***trxh2***		***trxo1***		***trxo1h2***	
**Metabolite**	**Fold change**	**Fold change**	**p-value**	**Fold change**	**p-value**	**Fold change**	**p-value**
**Soluble sugars**							
Arabinose	1.00±0.04	1.02±0.06	0.9880	0.92±0.04	0.4160	0.95±0.04	0.7740
Erythrose	1.00±0.10	1.05±0.04	0.7190	0.98±0.03	0.6920	0.90±0.08	1.0000
Fructose	1.00±0.05	0.98±0.05	0.8340	1.57±0.06	0.9910	1.36±0.20	0.9360
Fucose	1.00±0.03	1.02±0.04	0.9260	1.01±0.03	0.9960	1.02±0.04	0.6480
Galactose	1.00±0.12	1.87±0.26	0.9987	1.14±0.12	**0.0045**	1.46±0.15	0.0833
Glucose	1.00±0.07	0.98±0.04	0.9810	1.78±0.08	0.9990	1.34±0.12	0.9270
Maltose	1.00±0.05	1.03±0.03	0.8730	0.93±0.05	0.8700	0.96±0.04	0.8020
Mannose	1.00±0.04	1.21±0.03	0.9720	1.23±0.04	0.2570	1.23±0.07	0.9990
Psicose	1.00±0.07	0.97±0.04	**0.0041**	0.97±0.03	0.8362	1.06±0.07	0.1009
Raffinose	1.00±0.07	0.74±0.12	0.9981	1.04±0.16	**0.0010**	0.95±0.12	**0.0215**
Ribose	1.00±0.05	1.02±0.03	0.5519	1.01±0.04	0.0759	0.98±0.04	0.5521
2-deoxy-ribose	1.00±0.08	0.93±0.07	0.9360	0.89±0.05	0.7760	0.92±0.09	1.0000
Sorbose	1.00±0.06	0.80±0.03	0.5520	0.81±0.02	0.9690	0.85±0.08	1.0000
Sucrose	1.00±0.04	0.98±0.05	0.8250	0.89±0.01	0.3440	0.79±0.03	0.7520
Threose	1.00±0.09	1.06±0.06	0.9480	1.12±0.06	0.5970	1.04±0.04	0.8240
Trehalose	1.00±0.03	0.95±0.03	**0.0221**	0.97±0.05	**0.0077**	0.92±0.04	**0.0071**
Xylose	1.00±0.05	0.97±0.04	0.9790	0.88±0.05	0.9700	0.93±0.05	0.8320
**Sugar alcohols**							
Arabitol	1.00±0.11	1.17±0.14	0.3660	0.83±0.09	0.9920	1.01±0.18	0.9820
Erythritol	1.00±0.07	1.07±0.06	0.9710	0.98±0.07	0.9960	1.05±0.08	0.9770
Galactinol	1.00±0.11	0.94±0.07	0.8520	0.94±0.03	0.5810	0.93±0.05	0.7720
Galactitol	1.00±0.06	1.05±0.12	0.0503	1.24±0.11	0.0547	1.01±0.12	0.1513
Glycerol	1.00±0.06	0.90±0.02	0.9730	1.21±0.06	0.0620	0.90±0.08	**0.0010**
Glycerophosphoglycerol	1.00±0.08	0.95±0.05	0.9750	0.93±0.04	0.8500	1.00±0.09	0.9230

Myo-inositol	1.00±0.04	0.93±0.03	0.8260	1.02±0.03	0.3880	1.00±0.06	0.9210
Maltitol	1.00±0.06	0.94±0.05	0.6920	0.89±0.03	0.8930	0.94±0.06	0.3560
Threitol	1.00±0.09	1.05±0.17	0.9900	0.91±0.05	0.2520	0.93±0.07	0.8670
Xylitol	1.00±0.08	1.03±0.07	0.9500	0.82±0.06	0.2380	0.93±0.09	0.6230
**Amino acids**							
Alanine	1.00±0.03	0.92±0.03	0.2505	0.80±0.03	**0.0010**	0.69±0.04	**0.0010**
3-cyano-alanine	1.00±0.11	0.92±0.05	0.8520	1.00±0.05	1.0000	0.83±0.09	0.3250
Arginine	1.00±0.08	0.95±0.05	0.9500	0.85±0.05	0.4100	0.88±0.12	0.5860
Asparagine	1.00±0.09	0.95±0.08	0.9700	0.98±0.11	0.9990	0.98±0.12	0.9960
Aspartic acid	1.00±0.04	0.92±0.02	0.5860	0.90±0.04	0.4050	0.85±0.09	0.1470
2-aminobutanoic acid	1.00±0.06	1.16±0.07	0.2020	1.09±0.07	0.5830	1.08±0.05	0.6600
4-amino-3-hydroxybutanoic acid	1.00±0.10	0.94±0.05	0.9500	0.75±0.06	0.1610	0.84±0.13	0.4690
Cysteine	1.00±0.10	1.20±0.18	0.6060	0.89±0.10	0.8720	1.02±0.15	0.9980
Glutamic acid	1.00±0.04	0.90±0.04	0.4550	0.92±0.03	0.5350	0.90±0.08	0.3640
Glutamine	1.00±0.10	0.88±0.04	0.6480	1.00±0.08	1.0000	0.82±0.10	0.3100
Glycine	1.00±0.19	0.75±0.04	0.3740	0.85±0.03	0.6560	0.91±0.07	0.9030
Glycylglycine	1.00±0.10	0.93±0.06	0.9030	0.79±0.06	0.2290	0.92±0.11	0.8710
Homoserine-lactone	1.00±0.09	1.13±0.09	0.4520	1.05±0.04	0.9390	1.00±0.04	1.0000
Isoleucine	1.00±0.07	1.05±0.01	0.8360	1.02±0.05	0.9820	1.04±0.05	0.8980
Leucine	1.00±0.08	1.05±0.01	0.8810	1.10±0.06	0.5370	1.11±0.06	0.4350
Lysine	1.00±0.07	1.01±0.05	1.0000	0.97±0.07	0.9900	1.03±0.11	0.9830
Methionine	1.00±0.07	1.03±0.06	0.9800	0.93±0.04	0.7690	0.95±0.07	0.8690
Ornithine	1.00±0.20	1.21±0.19	0.7700	0.92±0.18	0.9800	1.06±0.17	0.9920
Ornithine-1,5-lactam	1.00±0.19	1.41±0.14	0.2300	1.04±0.13	0.9950	1.07±0.17	0.9790
Phenylalanine	1.00±0.04	0.98±0.03	0.9800	0.92±0.04	0.5260	0.91±0.06	0.4390
Proline	1.00±0.11	1.31±0.08	0.1073	1.46±0.09	**0.0091**	1.47±0.11	**0.0078**
Trans-4-hydroxy-proline	1.00±0.06	0.97±0.03	0.9610	1.00±0.03	1.0000	0.95±0.08	0.8790
Pyroglutamic acid	1.00±0.02	0.86±0.03	0.1510	0.94±0.05	0.7410	0.87±0.07	0.1970
Serine	1.00±0.04	0.96±0.04	0.9210	1.01±0.03	0.9990	0.97±0.07	0.9250
N-acetyl-serine	1.00±0.11	0.90±0.09	0.7660	0.84±0.07	0.4540	0.85±0.08	0.4770
O-acetyl-serine	1.00±0.04	0.99±0.03	0.9950	0.96±0.03	0.8380	0.92±0.06	0.4690
Threonine	1.00±0.04	0.99±0.02	0.9880	1.00±0.02	1.0000	0.96±0.05	0.8250
Tyrosine	1.00±0.10	1.02±0.11	0.9990	0.97±0.13	0.9940	1.05±0.12	0.9840
Valine	1.00±0.07	1.10±0.05	0.5170	1.04±0.06	0.9200	1.04±0.06	0.9330

**Organic acids**							
2-Piperidinecarboxylic acid	1.00±0.18	1.23±0.05	0.6125	0.97±0.16	0.9983	1.60±0.15	**0.0239**
Arabinonic acid	1.00±0.17	1.11±0.33	0.9600	0.69±0.14	0.5080	0.75±0.07	0.6470
Ascorbic acid	1.00±0.09	0.78±0.05	0.3040	0.72±0.09	0.1130	0.92±0.12	0.8710
Azelaic Acid	1.00±0.07	0.95±0.11	0.9250	1.06±0.07	0.8810	0.92±0.04	0.7530
2,4-dihydroxybutanoic acid	1.00±0.06	1.00±0.09	1.0000	0.94±0.04	0.8510	0.90±0.07	0.5520
3,5-dimethoxy-trans-cinnamic acid	1.00±0.04	1.03±0.11	0.9944	0.87±0.10	0.6284	0.70±0.12	0.0917
Dehydroascorbic acid dimer	1.00±0.15	0.94±0.22	0.9880	0.66±0.10	0.3090	1.12±0.16	0.8960
Furan-2-carboxylic acid	1.00±0.15	1.23±0.06	0.3520	1.20±0.09	0.4230	0.89±0.09	0.8200
Galactonic acid	1.00±0.08	1.01±0.09	1.0000	1.00±0.10	1.0000	0.96±0.08	0.9820
Galactaric acid	1.00±0.02	0.78±0.05	**0.0138**	0.79±0.04	**0.0129**	0.79±0.07	**0.0153**
Gluconic acid	1.00±0.08	1.01±0.09	1.0000	1.00±0.10	1.0000	0.96±0.08	0.9820
2-hydroxyglutaric acid	1.00±0.06	0.90±0.04	0.4720	0.90±0.03	0.4740	0.93±0.08	0.6760
Glyceric acid	1.00±0.08	0.93±0.08	0.8000	0.98±0.02	0.9930	0.93±0.06	0.7380
Gulonic acid	1.00±0.06	0.90±0.03	0.4680	0.88±0.06	0.3110	0.90±0.06	0.4480
Hexadecanoic acid	1.00±0.08	0.85±0.09	0.3342	0.75±0.06	**0.0439**	0.71±0.04	**0.0191**
2-ethylhexanoic acid	1.00±0.04	1.33±0.04	**0.0010**	1.17±0.04	0.0767	1.10±0.07	0.4275
Itaconic acid	1.00±0.10	1.12±0.09	0.8510	1.21±0.16	0.4990	0.93±0.12	0.9570
Maleic acid	1.00±0.11	1.16±0.11	0.7270	1.20±0.14	0.5420	1.02±0.13	0.9980
2-isopropylmalic acid	1.00±0.04	0.93±0.04	0.5954	0.87±0.02	0.1213	0.82±0.06	**0.0214**
2-methylmalic acid	1.00±0.08	1.02±0.07	0.9940	0.98±0.04	0.9920	1.00±0.07	1.0000
6-hydroxynicotinic acid	1.00±0.09	0.90±0.04	0.4550	0.88±0.02	0.2930	0.94±0.04	0.7760
Phosphoric acid	1.00±0.08	0.80±0.08	0.1440	1.18±0.04	0.1680	1.11±0.07	0.5600
Quinic acid	1.00±0.12	0.87±0.08	0.6240	0.79±0.08	0.2450	0.76±0.06	0.1560
Ribonic acid	1.00±0.07	0.96±0.07	0.9623	0.76±0.05	**0.0400**	0.77±0.07	**0.0495**
Shikimic acid	1.00±0.05	0.97±0.02	0.8950	0.93±0.02	0.4150	0.89±0.05	0.1470
Sinapic acid	1.00±0.10	0.96±0.10	0.9800	0.97±0.07	0.9940	0.89±0.11	0.7410
Threonic acid	1.00±0.06	0.95±0.02	0.7343	0.82±0.02	**0.0162**	0.82±0.05	**0.0191**
**TCA cycle intermediates**							
Aconitic acid	1.00±0.09	1.00±0.09	1.0000	0.72±0.07	0.0596	0.80±0.07	0.2311
Citric acid	1.00±0.06	0.95±0.05	0.8310	0.98±0.04	0.9880	1.06±0.04	0.7010
Fumaric acid	1.00±0.05	0.92±0.06	0.6680	0.99±0.02	0.9990	0.96±0.08	0.9030
2-oxoglutaric acid	1.00±0.08	0.93±0.07	0.8750	0.99±0.07	1.0000	1.05±0.10	0.9430
Malic acid	1.00±0.04	0.97±0.04	0.9650	0.98±0.04	0.9890	0.95±0.09	0.9040

Pyruvic acid	1.00±0.08	1.08±0.09	0.8810	1.11±0.10	0.7110	0.90±0.10	0.7890
Succinic acid	1.00±0.08	1.03±0.04	0.9860	1.00±0.05	1.0000	1.04±0.07	0.9540
**Phosphate intermediates**							
Fructose-6-phosphate	1.00±0.08	0.81±0.02	0.0651	0.81±0.02	**0.0419**	0.75±0.05	**0.0074**
Glucose-6-phosphate	1.00±0.07	0.84±0.04	0.1100	0.87±0.02	0.1820	0.86±0.06	0.1340
Glyceric acid-3-phosphate	1.00±0.14	0.84±0.06	0.7000	1.10±0.12	0.8880	0.97±0.11	0.9960
Glycerol-3-phosphate	1.00±0.03	0.91±0.04	0.3330	1.01±0.02	0.9970	0.98±0.07	0.9810
**Miscellaneous**							
3,6-dimethyl-2,5-Piperazinedione	1.00±0.15	1.05±0.11	0.9940	1.39±0.20	0.2700	1.10±0.19	0.9560
1,2,4-benzenetriol	1.00±0.12	1.27±0.10	0.4060	1.19±0.17	0.6440	1.06±0.14	0.9760
Erythronic acid-1,4-lactone	1.00±0.05	0.87±0.03	0.2240	1.05±0.03	0.8370	1.00±0.07	1.0000
Ethanolamine	1.00±0.27	0.64±0.03	0.5547	2.17±0.22	**0.0028**	1.21±0.23	0.8312
N-acetylGalactosamine	1.00±0.07	1.01±0.04	1.0000	1.04±0.04	0.9390	1.05±0.06	0.9000
2-amino-2-deoxy-galactose	1.00±0.21	0.69±0.05	0.3600	1.23±0.15	0.5740	0.92±0.11	0.9570
Glucoheptonic acid-1,4-lactone	1.00±0.05	0.92±0.03	0.5630	1.02±0.05	0.9740	0.88±0.06	0.1900
Gluconic acid-1,4-lactone	1.00±0.15	0.78±0.04	0.3230	0.90±0.04	0.7960	0.87±0.10	0.6700
Gluconic acid-1,5-lactone	1.00±0.08	1.05±0.10	0.9100	0.84±0.02	0.2730	1.00±0.06	1.0000
Glucuronic acid-3,6-lactone	1.00±0.07	1.19±0.08	0.7131	1.68±0.23	**0.0105**	1.28±0.14	0.4030
Phosphoric acid monomethyl ester	1.00±0.07	0.87±0.04	0.3757	0.80±0.05	0.0729	0.84±0.07	0.1711
Putrescine	1.00±0.06	0.86±0.01	0.0523	0.90±0.02	0.2110	0.85±0.04	**0.0334**
Ribonic acid-1,4-lactone	1.00±0.08	1.09±0.08	0.7300	0.99±0.07	1.0000	1.10±0.07	0.6880
Spermidine	1.00±0.08	1.02±0.03	0.9920	1.07±0.03	0.8080	1.06±0.10	0.8980
Uracil	1.00±0.12	0.98±0.08	1.0000	1.00±0.17	1.0000	0.77±0.12	0.4730
Urea	1.00±0.15	0.60±0.02	**0.0105**	0.73±0.03	0.0775	0.67±0.04	**0.0307**
Uric acid	1.00±0.15	1.05±0.10	0.9810	0.91±0.08	0.8630	0.97±0.09	0.9950

**Phase**	**Arabidopsis leaves (LL)**						
**Genotype**	**Col-0**	***trxh2***		***trxo1***		***trxo1h2***	
**Metabolite**	**Fold change**	**Fold change**	**p-value**	**Fold change**	**p-value**	**Fold change**	**p-value**
**Soluble sugars**							
Arabinose	1.00±0.05	1.09±0.08	0.7180	0.88±0.04	0.5320	1.02±0.11	0.9950
Erythrose	1.00±0.05	1.36±0.02	0.7740	1.19±0.09	0.9190	1.13±0.13	0.9930
Fructose	1.00±0.05	1.08±0.06	0.2080	1.10±0.08	0.9670	0.90±0.02	0.9430
Fucose	1.00±0.02	1.08±0.05	**0.0301**	0.95±0.03	0.3430	1.02±0.04	0.6280
Galactose	1.00±0.22	0.62±0.07	0.6880	1.25±0.31	0.5080	0.62±0.05	0.5120
Glucose	1.00±0.04	1.25±0.07	0.3720	1.11±0.06	0.7420	1.05±0.03	0.9850
Maltose	1.00±0.05	0.92±0.05	0.6800	1.09±0.12	0.9850	0.83±0.03	0.6200
Mannose	1.00±0.03	1.30±0.06	0.1170	1.19±0.04	0.9870	1.25±0.03	0.4410
Psicose	1.00±0.02	1.10±0.04	0.4110	1.01±0.04	0.6990	1.05±0.03	0.4050
Raffinose	1.00±0.34	1.15±0.19	**0.0136**	1.17±0.31	0.3971	0.91±0.15	0.8422
Ribose	1.00±0.03	1.11±0.05	0.3310	0.96±0.04	0.9990	1.03±0.04	0.4670
2-deoxy-ribose	1.00±0.06	1.07±0.11	0.2170	0.91±0.08	0.9630	0.98±0.06	0.1330
Sorbose	1.00±0.12	0.98±0.12	0.7580	0.78±0.04	0.3020	0.95±0.10	0.8020
Sucrose	1.00±0.04	0.97±0.05	0.9160	0.98±0.05	0.4270	0.89±0.04	0.1760
Threose	1.00±0.09	1.19±0.10	0.7800	1.06±0.10	0.7050	1.10±0.09	0.2870
Trehalose	1.00±0.04	1.08±0.07	**0.0010**	0.96±0.06	**0.0158**	1.06±0.01	**0.0016**
Xylose	1.00±0.04	1.09±0.03	0.1650	0.94±0.05	0.9910	0.97±0.05	0.6280
**Sugar alcohols**							
Arabitol	1.00±0.18	1.20±0.23	0.9480	0.87±0.14	0.9370	1.06±0.17	0.9880
Erythritol	1.00±0.04	1.15±0.04	0.2310	0.97±0.06	0.8380	1.04±0.08	0.9380
Galactinol	1.00±0.03	1.05±0.04	0.8970	1.02±0.04	0.7810	1.06±0.04	0.9960
Galactitol	1.00±0.07	1.39±0.13	0.9980	1.05±0.18	0.2960	1.24±0.08	0.9740
Glycerol	1.00±0.06	1.25±0.11	0.9360	0.98±0.10	0.9790	1.21±0.15	0.3040
Glycerophosphoglycerol	1.00±0.14	1.27±0.07	0.6730	1.06±0.12	1.0000	1.32±0.10	0.8510
Myo-inositol	1.00±0.05	1.05±0.03	0.4140	1.10±0.05	0.9470	1.05±0.04	0.8060
Maltitol	1.00±0.04	0.96±0.04	0.6270	0.89±0.10	0.8920	0.84±0.04	0.7220
Threitol	1.00±0.06	1.15±0.11	0.6840	0.99±0.08	0.7370	1.10±0.16	1.0000
Xylitol	1.00±0.10	1.17±0.08	0.3680	1.15±0.18	0.6910	0.98±0.11	0.9220

**Amino acids**							
Alanine	1.00±0.03	1.01±0.01	0.9945	0.89±0.02	0.3802	0.78±0.09	**0.0276**
3-cyano-alanine	1.00±0.07	1.01±0.08	1.0000	1.03±0.04	0.9870	0.85±0.14	0.5230
Arginine	1.00±0.07	1.35±0.10	0.1620	1.09±0.09	0.9210	1.10±0.19	0.8930
Asparagine	1.00±0.21	1.12±0.08	0.8800	0.90±0.12	0.9340	1.08±0.17	0.9650
Aspartic acid	1.00±0.05	1.09±0.05	0.6760	1.00±0.04	1.0000	0.97±0.11	0.9750
2-aminobutanoic acid	1.00±0.08	1.17±0.05	0.2620	0.99±0.07	1.0000	1.02±0.08	0.9950
4-amino-3-hydroxybutanoic acid	1.00±0.10	1.17±0.13	0.6070	0.91±0.09	0.9170	1.08±0.13	0.9350
Cysteine	1.00±0.14	1.14±0.22	0.8950	0.81±0.14	0.7920	1.04±0.17	0.9970
Glutamic acid	1.00±0.06	1.07±0.04	0.7800	1.00±0.04	1.0000	0.96±0.09	0.9340
Glutamine	1.00±0.10	1.14±0.14	0.7690	1.21±0.10	0.5190	0.93±0.15	0.9540
Glycine	1.00±0.12	1.50±0.13	**0.0133**	1.34±0.10	0.1097	1.45±0.06	**0.0268**
Glycylglycine	1.00±0.09	1.35±0.08	0.0681	1.04±0.09	0.9806	1.26±0.13	0.1930
Homoserine-lactone	1.00±0.05	1.19±0.04	0.1390	1.05±0.09	0.8970	1.08±0.06	0.6890
Isoleucine	1.00±0.05	1.25±0.07	**0.0167**	1.11±0.05	0.3970	1.13±0.05	0.2914
Leucine	1.00±0.04	1.17±0.16	0.4620	1.07±0.05	0.9180	1.14±0.05	0.5770
Lysine	1.00±0.09	1.25±0.11	0.2610	1.03±0.13	0.9920	1.09±0.07	0.8790
Methionine	1.00±0.07	1.15±0.04	0.3330	0.99±0.05	0.9980	0.96±0.10	0.9670
Ornithine	1.00±0.19	1.31±0.12	0.5700	0.90±0.21	0.9680	1.12±0.24	0.9460
Ornithine-1,5-lactam	1.00±0.14	1.26±0.12	0.4450	1.02±0.18	0.9990	1.14±0.12	0.8180
Phenylalanine	1.00±0.05	1.12±0.04	0.3030	0.97±0.06	0.9570	1.06±0.05	0.7620
Proline	1.00±0.04	1.25±0.03	**0.0296**	1.41±0.08	**0.0010**	1.16±0.08	0.2318
Trans-4-hydroxy-proline	1.00±0.04	1.07±0.02	0.7880	1.11±0.06	0.4610	0.97±0.09	0.9690
Pyroglutamic acid	1.00±0.11	0.99±0.03	1.0000	1.02±0.05	0.9960	0.90±0.05	0.5340
Serine	1.00±0.06	1.06±0.05	0.7540	1.07±0.05	0.6740	0.98±0.05	0.9880
N-acetyl-serine	1.00±0.14	1.06±0.13	0.9780	0.90±0.13	0.8940	0.86±0.10	0.7590
O-acetyl-serine	1.00±0.04	0.68±0.01	**0.0010**	0.69±0.03	**0.0010**	0.76±0.05	**0.0010**
Threonine	1.00±0.03	1.13±0.03	0.0504	1.10±0.02	0.1344	1.03±0.05	0.8187
Tyrosine	1.00±0.15	1.88±0.64	0.2370	0.97±0.17	1.0000	1.19±0.08	0.9670
Valine	1.00±0.10	1.23±0.03	**0.0346**	1.10±0.05	0.4893	1.10±0.05	0.4936
**Organic acids**							
2-Piperidinecarboxylic acid	1.00±0.14	1.01±0.05	1.0000	1.03±0.07	0.9940	0.96±0.13	0.9840
Arabinonic acid	1.00±0.13	0.76±0.12	0.3400	0.92±0.12	0.9140	0.65±0.07	0.1070
Ascorbic acid	1.00±0.09	0.67±0.25	0.6180	1.49±0.30	0.3200	0.64±0.18	0.5630

Azelaic Acid	1.00±0.21	1.07±0.17	0.9750	0.80±0.06	0.7060	1.13±0.15	0.8900
2,4-dihydroxybutanoic acid	1.00±0.11	1.16±0.09	0.5980	1.06±0.13	0.9650	0.96±0.09	0.9900
3,5-dimethoxy-trans-cinnamic acid	1.00±0.16	1.16±0.10	0.8880	1.44±0.33	0.3140	1.08±0.08	0.9850
Dehydroascorbic acid dimer	1.00±0.23	1.12±0.40	0.9890	1.32±0.28	0.8460	1.20±0.37	0.9530
Furan-2-carboxylic acid	1.00±0.21	1.23±0.08	0.4580	1.16±0.09	0.7170	0.88±0.12	0.8330
Galactonic acid	1.00±0.23	1.04±0.09	0.9960	1.14±0.14	0.8130	0.90±0.08	0.9240
Galactaric acid	1.00±0.04	1.10±0.11	0.6790	1.02±0.07	0.9930	1.04±0.03	0.9600
Gluconic acid	1.00±0.23	1.04±0.09	0.9960	1.14±0.14	0.8130	0.90±0.08	0.9240
2-hydroxyglutaric acid	1.00±0.03	1.05±0.02	0.7720	0.99±0.05	0.9980	0.91±0.05	0.2770
Glyceric acid	1.00±0.04	1.00±0.03	1.0000	1.03±0.04	0.9250	1.12±0.07	0.2640
Gulonic acid	1.00±0.04	1.07±0.02	0.4590	0.94±0.04	0.5040	0.94±0.05	0.5560
Hexadecanoic acid	1.00±0.10	0.92±0.10	0.8580	0.83±0.07	0.4250	0.77±0.09	0.2190
2-ethylhexanoic acid	1.00±0.13	1.42±0.06	**0.0169**	1.14±0.11	0.6157	1.04±0.08	0.9863
Itaconic acid	1.00±0.21	0.85±0.11	0.7860	0.82±0.13	0.7220	0.67±0.12	0.2760
Maleic acid	1.00±0.18	1.13±0.08	0.8670	1.06±0.18	0.9840	0.90±0.15	0.9390
2-isopropylmalic acid	1.00±0.05	0.89±0.04	0.1741	0.77±0.03	**0.0015**	0.86±0.04	0.0537
2-methylmalic acid	1.00±0.06	1.21±0.11	0.2760	1.05±0.09	0.9680	1.01±0.08	1.0000
6-hydroxynicotinic acid	1.00±0.05	0.91±0.04	0.3257	0.83±0.03	**0.0276**	0.88±0.04	0.1584
Phosphoric acid	1.00±0.04	1.17±0.21	0.7040	1.15±0.06	0.7710	1.28±0.12	0.3350
Quinic acid	1.00±0.15	1.11±0.11	0.7870	0.96±0.08	0.9830	1.05±0.03	0.9680
Ribonic acid	1.00±0.03	0.93±0.03	0.3920	0.90±0.05	0.1894	0.81±0.03	**0.0051**
Shikimic acid	1.00±0.03	1.10±0.04	0.2060	0.96±0.04	0.8420	1.03±0.04	0.9150
Sinapic acid	1.00±0.12	1.28±0.08	0.1520	0.97±0.10	0.9900	1.17±0.10	0.5330
Threonic acid	1.00±0.03	1.20±0.06	0.1350	1.01±0.08	1.0000	1.07±0.08	0.8110
**TCA cycle intermediates**							
Aconitic acid	1.00±0.06	0.96±0.08	0.9610	0.90±0.08	0.5980	0.84±0.02	0.2210
Citric acid	1.00±0.06	1.27±0.06	**0.0055**	1.31±0.04	**0.0015**	1.23±0.04	**0.0159**
Fumaric acid	1.00±0.05	1.13±0.04	0.2500	1.08±0.05	0.5850	0.83±0.07	0.1030
2-oxoglutaric acid	1.00±0.08	1.31±0.11	0.1141	1.08±0.10	0.8892	1.36±0.09	0.0579
Malic acid	1.00±0.08	1.17±0.03	0.3300	1.16±0.07	0.3620	0.97±0.11	0.9870
Pyruvic acid	1.00±0.10	1.23±0.12	0.3790	1.14±0.12	0.7000	0.97±0.11	0.9940
Succinic acid	1.00±0.06	1.17±0.03	0.1740	1.11±0.09	0.4970	1.04±0.05	0.9260
**Phosphate intermediates**							
Fructose-6-phosphate	1.00±0.11	1.55±0.08	**0.0011**	1.19±0.11	0.3591	1.40±0.05	**0.0165**

Glucose-6-phosphate	1.00±0.11	1.39±0.06	**0.0141**	1.09±0.11	0.7857	1.37±0.05	**0.0199**
Glyceric acid-3-phosphate	1.00±0.24	1.12±0.15	0.9160	1.03±0.15	0.9980	0.90±0.09	0.9370
Glycerol-3-phosphate	1.00±0.07	1.23±0.05	0.1680	1.13±0.13	0.5820	1.19±0.06	0.3070
**Miscellaneous**							
3,6-dimethyl-2,5-Piperazinedione	1.00±0.21	1.35±0.21	0.5370	1.24±0.24	0.7740	1.20±0.18	0.8490
1,2,4-benzenetriol	1.00±0.17	1.20±0.22	0.8280	1.02±0.23	1.0000	0.85±0.19	0.9180
Erythronic acid-1,4-lactone	1.00±0.05	0.93±0.03	0.4710	0.94±0.05	0.5970	0.96±0.03	0.8710
Ethanolamine	1.00±0.10	1.68±0.21	0.0915	0.97±0.20	0.9989	2.04±0.27	**0.0077**
N-acetylGalactosamine	1.00±0.03	1.14±0.06	0.1430	0.98±0.05	0.9800	1.04±0.04	0.9060
2-amino-2-deoxy-galactose	1.00±0.28	1.31±0.24	0.6580	1.16±0.23	0.9280	1.01±0.13	1.0000
Glucoheptonic acid-1,4-lactone	1.00±0.05	1.24±0.06	**0.0162**	1.01±0.04	0.9956	1.09±0.06	0.5559
Gluconic acid-1,4-lactone	1.00±0.14	1.51±0.15	**0.0249**	1.00±0.11	1.0000	1.51±0.09	**0.0233**
Gluconic acid-1,5-lactone	1.00±0.02	1.28±0.12	0.0681	1.06±0.05	0.9260	1.12±0.08	0.6166
Glucuronic acid-3,6-lactone	1.00±0.04	1.24±0.08	0.2100	0.92±0.13	0.8600	1.00±0.09	1.0000
Phosphoric acid monomethyl ester	1.00±0.05	1.13±0.07	0.3060	0.91±0.04	0.5880	1.12±0.07	0.3470
Putrescine	1.00±0.05	1.04±0.03	0.8450	0.90±0.04	0.1960	0.99±0.04	0.9970
Ribonic acid-1,4-lactone	1.00±0.14	1.21±0.09	0.2540	0.90±0.07	0.7460	1.20±0.05	0.2940
Spermidine	1.00±0.09	1.38±0.07	0.1090	1.15±0.18	0.7130	1.24±0.12	0.4020
Uracil	1.00±0.18	1.00±0.12	1.0000	0.97±0.17	0.9980	0.78±0.10	0.5710
Urea	1.00±0.06	0.92±0.10	0.8040	0.76±0.09	0.1060	1.56±0.05	**0.0010**
Uric acid	1.00±0.11	1.23±0.05	0.0802	1.11±0.04	0.6201	1.16±0.07	0.3027

Table S3 Prediction of conserved cysteine in enzymes of AsA-GSH cycle

Name	UniPort ID	Locus	Homologues	Conserved	Cys position	Cys score	*p* value	Subcelular localization
**Ascorbate Peroxidase**								
APX1	Q05431	At1g07890	20	2 of 5	Cys1: 19	0.1500	9.72E-01	Cytosol
					Cys2: 32	**0.6000**	3.37E-03	
					Cys3: 49	0.1000	9.95E-01	
					Cys4: 138	0.1000	9.95E-01	
					Cys5: 168	**0.7500**	1.50E-05	
APX2	Q1PER6	At3g09640	20	2 of 3	Cys1: 20	0.2000	8.65E-01	Cytosol
					Cys2: 33	**0.6000**	1.40E-03	
					Cys3: 168	**0.7000**	3.60E-05	
APX3	Q42564	At4g35000	20	2 of 3	Cys1: 30	**0.6000**	9.17E-03	Peroxisome
					Cys2: 79	0.3000	7.23E-01	
					Cys3: 123	**0.5500**	3.03E-02	
APX4	A0A1P8B8W6	At4g09010	17	1 of 2	Cys1: 138	**0.5294**	6.17E-01	Microsome
					Cys2: 260	0.0588	1.00E+00	
APX5	Q7XZP5	At4g35970	20	1 of 3	Cys1: 29	**0.7000**	5.67E-04	Microsome
					Cys2: 78	0.3000	7.25E-01	
					Cys3: 271	0.0500	1.00E+00	
APX6	Q8GY91	At4g32320	20	2 of 6	Cys1: 16	0.2000	9.97E-01	Cytosol
					Cys2: 26	0.3000	9.64E-01	
					Cys3: 56	0.0500	1.00E+00	

					Cys4: 190	**0.5000**	4.70E-01	
					Cys5: 226	0.1000	1.00E+00	
					Cys6: 294	**0.6000**	1.59E-01	
tAPX	Q42593	At1g77490	20	2 of 4	Cys1: 13	0.1000	1.00E+00	Thylokoid
					Cys2: 78	0.3000	9.35E-01	
					Cys3: 102	**0.7000**	1.14E-02	
					Cys4: 202	**0.7500**	2.83E-03	
sAPX	Q42592	At4g08390	20	2 of 2	Cys1: 123	**0.7000**	5.69E-03	Stroma
					Cys2: 223	**0.7500**	1.29E-03	
**Monodehydroascorbate Reductase**								
MDHAR1	Q9LFA3	At3g52880	19	2 of 3	Cys1: 68	0.0526	1.00E+00	Peroxisome
					Cys2: 69	**0.7368**	3.35E-04	
					Cys3: 198	**0.5263**	7.65E-02	
MDHAR2	Q93WJ8	At5g03630	19	1 of 1	Cys1: 199	**0.5263**	1.05E-01	Cytosol
MDHAR3	Q9SR59	At3g09940	19	1 of 1	Cys1: 70	**0.7368**	9.55E-04	Cytosol
MDHAR4	Q9LK94	At3g27820	19	3 of 5	Cys1: 34	0.4737	2.60E-01	Peroxisome
					Cys2: 68	**0.7368**	8.55E-04	
					Cys3: 142	0.4737	2.60E-01	
					Cys4: 177	**0.6316**	1.68E-02	
					Cys5: 197	**0.5789**	5.17E-02	
MDHAR6	P92947	At1g63940	19	2 of 5	Cys1: 25	0.1579	9.97E-01	Plastid
					Cys2: 90	0.4211	5.68E-01	
					Cys3: 126	**0.7368**	2.59E-03	

					Cys4: 181	**0.6316**	3.67E-02	
					Cys5: 379	0.4737	3.74E-01	
**Dehydroascorbate reductase**								
DHAR1	Q9FWR4	At1g19570	17	1 of 2	Cys1: 6	0.4118	3.40E-01	Peroxisome
					Cys2: 20	**0.9412**	1.25E-08	
DHAR2	Q9FRL8	At1g75270	17	1 of 2	Cys1: 6	0.2353	9.15E-01	Cytosol
					Cys2: 20	**0.9412**	1.31E-08	
DHAR3	Q8LE52	At5g16710	15	1 of 4	Cys1:28	0.0000	1.00E+06	Plastid
					Cys2: 52	0.4000	4.82E-02	
					Cys3: 66	**0.9333**	2.09E-10	
					Cys4: 69	0.3333	1.48E-01	
**Glutathione Reductase**								
GR1	P48641	At3g24170	19	5 of 8	Cys1: 54	**0.5789**	2.78E-02	Cytosol Peroxisome
					Cys2: 73	**0.9474**	9.70E-09	
					Cys3: 78	**0.9474**	9.70E-09	
					Cys4: 355	**0.5263**	7.66E-02	
					Cys5: 375	0.4737	1.73E-01	
					Cys6: 379	0.3684	5.18E-01	
					Cys7: 441	0.3684	5.18E-01	
					Cys8: 458	**0.6842**	1.91E-03	
GR2	P42770	At3g54660	19	3 of 9	Cys1: 22	0.0526	1.00E+00	Plastid
					Cys2: 73	0.0000	1.00E+06	

Cys3: 116	**0.6842**	1.02E-02
Cys4: 135	**0.9474**	1.69E-07
Cys5: 140	**0.9474**	1.69E-07
Cys6: 284	0.0000	1.00E+06
Cys7: 433	0.1579	9.97E-01
Cys8: 486	0.3684	7.41E-01
Cys9: 498	0.4737	3.64E-01

The protein information obtained from Uniport was used to predict the redox-reactive Cys via ConCysFind. The information shown in the table include UniportID, the number of identified homologous genes among 21 species, positions and confident scores of identified Cys as well as the subcellular localizations. The information of subcellular localization was obtained from literatures or predicted via SUBA. Cys scores over than 0.5 are marked green color indicating higher confidentiality.
